# EDA photochemistry using continuous flow

**DOI:** 10.1007/s41981-025-00362-3

**Published:** 2025-09-18

**Authors:** Samuel L. Nickels, Ai-Lan Lee, Filipe Vilela

**Affiliations:** 1 School of Engineering and Physical Sciences, Institute of Chemical Sciences, Heriot Watt University, Edinburgh, EH14 4AS UK; 2 EaStCHEM School of Chemistry, University of Edinburgh, David Brewster Road, Edinburgh, EH9 3FJ UK

**Keywords:** Electron donor-acceptor complexes, Flow photochemistry, Organic synthesis, Photocatalyst-free

## Abstract

In recent years, electron donor–acceptor (EDA) complexes have emerged as sustainable, cost-effective, and inherently safer alternatives to traditional transition metal-based photocatalysts in photochemical processes. Formed via the association of neutral electron-rich and electron-deficient species, EDAs offer an environmentally benign route to radical generation across a broad spectrum of reactions. Concurrently, flow chemistry has gained prominence as a burgeoning area of scientific inquiry, enhancing reaction control, safety, mixing efficiency, and, critically, light penetration. In this mini review, we highlight recent works that have explored the use of EDA photochemistry with flow methodologies, while assessing both the promising practical advantages and current limitations in bringing these two fields together.

**Graphical Abstract:**

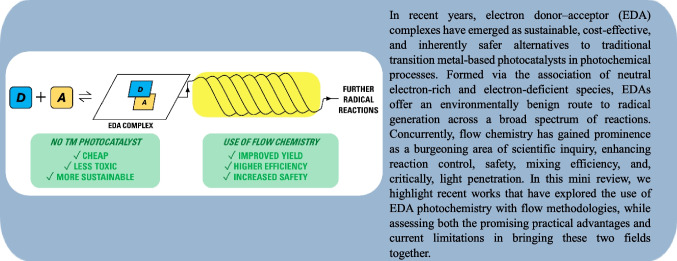

## Introduction

### Background

The growing concerns surrounding the environment, and the global energy crisis have driven an urgent search for more sustainable and efficient methodologies for conducting chemical reactions [1–10]. Traditional energy sources, such as fossil fuels, have not only contributed to environmental degradation and climate change but are also finite and unsustainable in the long run. Chemical reactions often rely on heat as the primary energy source to overcome activation energy [11–15], requiring significant fossil fuel consumption and contributing to greenhouse gas emissions. As a result, the scientific community is increasingly turning to alternative energy sources to drive chemical transformations.

Photochemistry, the study of chemical reactions driven by light, offers a promising solution [16–22] by using light to access high-energy intermediates without requiring thermal input to initiate reactions, instead utilising photons as “traceless and green reagents” [23]. The first organic synthetic photochemical reaction was discovered by Trommsdorff in 1834 [24], employing sunlight as a source of photons to burst crystals of α-santonin [25]. However, progress within the field was, at first, mostly limited to the research of external photocatalysts [18, 20, 26–28].

Many photocatalysts are typically transition metal based complexes, using metals such as nickel [29], iridium [18, 29], ruthenium [18, 30], palladium [18] and copper [18]. Due to their affinity towards strong visible light absorption, these photocatalysts can catalyse and stimulate radical formation and mechanisms via single-electron transfer (SET) [18, 29, 30]. However, transition metal complexes are not only expensive, but also toxic and are now becoming ever more unsustainable [31–33]. Therefore, it is imperative for metal- and even photocatalyst-free techniques to be found to enable greener and cheaper pathways for light-mediated organic reactions.

To avoid the toxic, expensive and unsustainability of metal-based photocatalysts, there has been increasing interest in the development of molecular organic photocatalysts[34]. These organic photocatalysts can be synthesised from renewable materials, are much more soluble in organic solvents and are generally more chemically stable due to the lack of metal-coordination effects [35–37], however, these molecular organic photocatalysts come with limitations of scope as well as selectivity [34], which has inspired a search for other solutions, including photocatalyst-free photochemistry. Since 2008, the specific research field of organic photochemical synthesis activated by visible light has been driven by MacMillan [18, 20, 38, 39], Stephenson [21, 40, 41], Yoon [22, 30, 42], Nicewicz [26, 43–46] and Melchiorre [29, 47–49], amongst others.

**Scheme 1:**
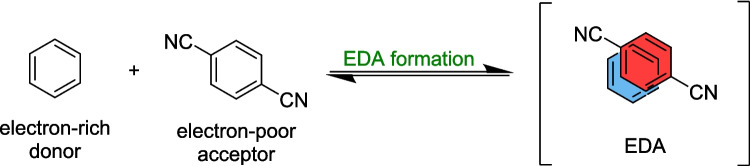
The formation of an electron donor–acceptor (EDA) complex was observed. Foster [50] found that the EDA formed here exhibited a significant bathochromic shift compared to the starting substrates

The most common form of a photocatalyst-free light-mediated organic reaction pathway involves an electron donor-acceptor, or EDA, complex, defined as the association of an electron-rich, or -donor, substrate and an electron-poor, or -acceptor, substrate, creating a ground-state aggregate (Scheme 1). This aggregate absorbs light at a different wavelength, typically in the visible range, to that of either of the two substates [50].

First described in the early 1950’s by Robert Mulliken [51–53], EDA complexes have, in recent years, become ever more present in the field of sustainable photochemical synthesis, since the presence of suitable EDA complexes can negate the requirement for an exogenous photocatalyst, while maintaining mild reaction conditions. Therefore, EDA complex photochemistry has emerged as a powerful, inexpensive and efficient tool in the field of light-driven radical chemistry [54–56].

This mini review aims to provide insight into studies that have employed EDA photochemistry within flow apparatus, highlighting the key benefits and drawbacks of the application of flow chemistry in EDA photochemistry and putting a spotlight on interesting recent advances in the field. EDA complexes have been subject to many excellent reviews on their reactivity and mechanisms, and we direct the readers to those of Stephenson [54], Melchiorre [55], Pitre [56], Bugaenko & Karchava [57] and Barata-Vallejo & Postigo [58]. Much like the exploration within the field of EDA complexes, there have been many outstanding reviews for photochemistry in flow, and we highlight those of Rehm [59], Xie & Zhao [60], Vilela & Lee [61], Politano & Oksdath-Mansilla [62], Gilmour & Seeburger [63] and Noël [64–66]. This mini review will instead focus specifically on the utilisation of EDA photochemistry within a flow reactor manifold, emphasising the main benefits as well as the key drawbacks within this field, with the hope that this will spark further studies to investigate this area.

### EDA photochemistry

**Fig. 1:**
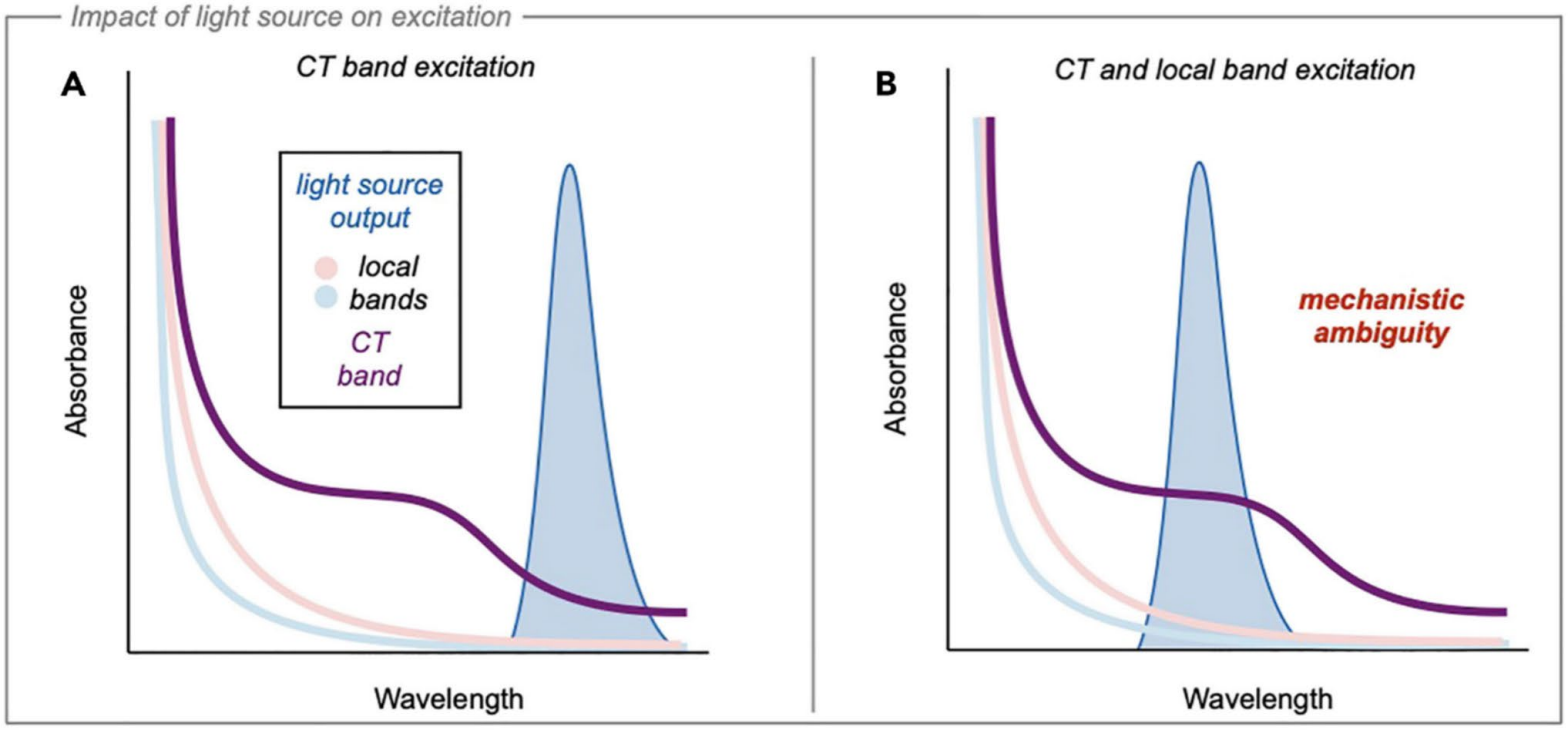
Simulated overlay of the light source with the local and CT bands **A**) overlap of the light source and CT band **B**) Overlap of the light source with the CT and local bands, causing excitation. *Reproduced from Wortman & Stephenson *[54]* with permission from Elsevier, Copyright Clearance Centre *[68]

In the early 1950’s, Robert Mulliken [51–53] described the process of electron transfer, which is a key driving force behind many organic syntheses, especially those including photochemical, electrochemical, and enzymatic reactions. He proposed that quantum mechanical theory could explain the experimentally observed phenomena of a bathochromic shift from the absorbances of free donor and acceptor species. This broad absorption peak was defined as the charge-transfer (CT) band [50]. This band is located within the visible region of the UV-vis (ultraviolet-visible) spectrum [67], which makes the use of EDAs especially suitable for photochemical reactions, especially since the separate donor and acceptor components of an EDA may not absorb visible light separately, but the combination of the both allows for this transition (Figure 1).

**Scheme 2:**
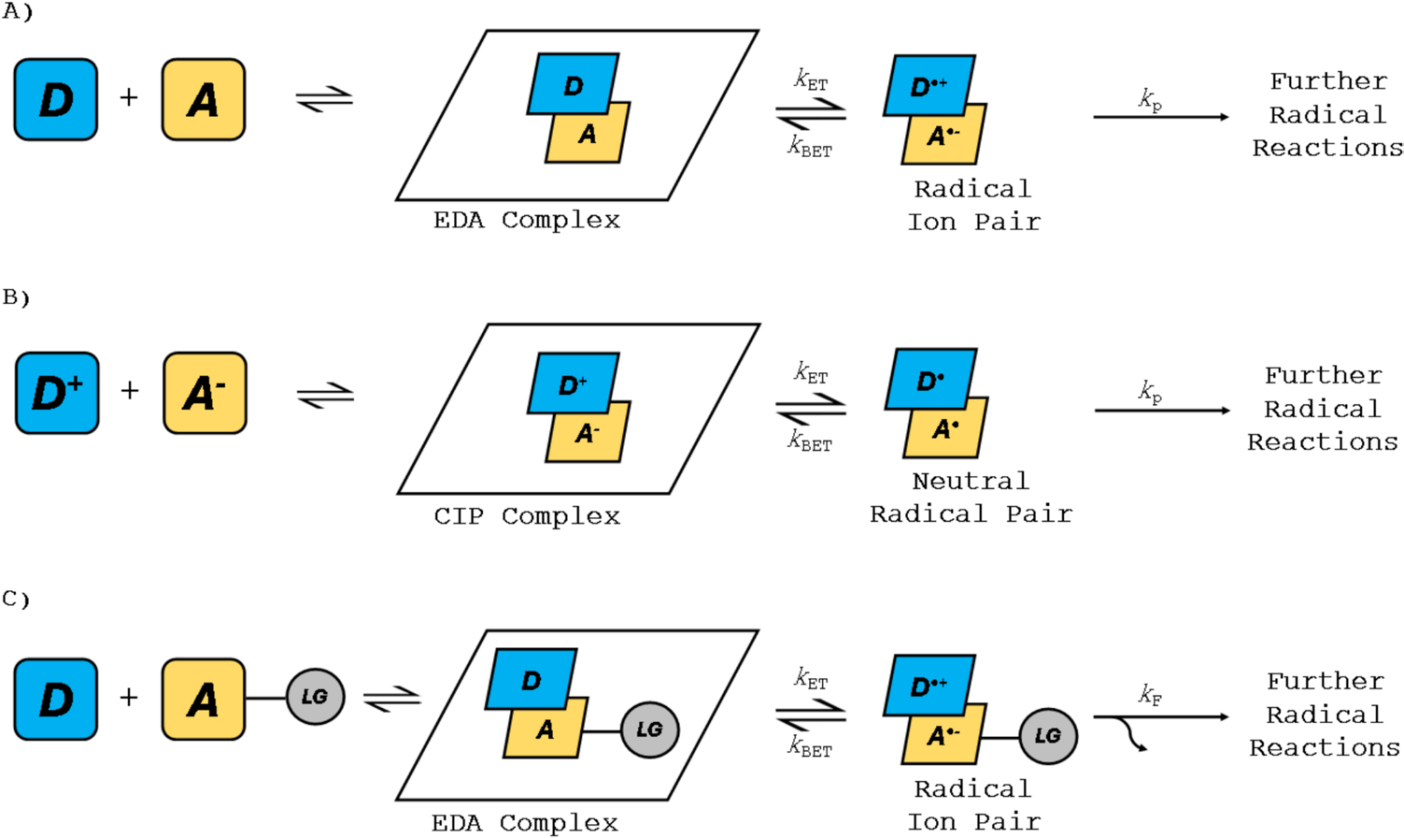
The mechanisms of EDA complexation **A**) The conventional EDA complexation **B**) The conventional CIP complexation. **C**) The irreversible fragmentation of a leaving group from an EDA to access radicals for further reactions. **D** is the donor, and A is the acceptor, and LG is a leaving group

The electronic transition of a single electron from the ground-state complex to an excited state complex corresponds to this CT band [69], therefore making the transition a light-promoted single electron transfer (SET) within a complex. This ultimately leads to the production of a pair of radical intermediates (Scheme 2A) that can form at mild conditions at room temperature [70].

Although the formation of an EDA complexes often causes subtle differences in NMR chemical shift and vibrational frequencies explored in IR-spectroscopy, the most common way the formation of an EDA complex can be assessed is via UV-vis spectroscopy [49, 54, 71–76], where an EDA can be identified by appearance of a redshift, or bathochromic shift in the absorption band of the mixture of the donor and acceptor organic compounds. A visible colour change can also be seen upon mixing of the two components, while fluorescence analysis can be used to investigate the excitation wavelength and emission intensity associated with the newly formed EDA complex [47, 77–81].

UV-vis spectroscopy can also be used to identify the appearance of new charge-transfer (CT) absorption band (Figure 1) associated with single electron transfer (SET) from donor to acceptor upon excitation. This band is often distinct from the individual absorption spectra of the components, and will often appear in the visible or near-UV region, even if the individual acceptor and donor molecules do not absorb there [71, 74, 75].

Additionally, a Job plot, plotting the absorbance against the mole fraction of the two donor or acceptor molecules [82], has occasionally been used to identify to determine the molecular composition of the EDA complex [83–85], as has been done in organometallic chemistry [86, 87], however, this method cannot solely identify the presence of an EDA-complex, and is often used in tandem with UV-vis spectroscopy.

EDAs are formed from a pair of neutral donor and acceptor species, resulting in the formation of a radical ion pair upon irradiation (Scheme 2A). However, when one or both species are charged, such as an anion and cation, this instead leads to the formation of a contact ion pair (CIP) [88, 89]. This follows a similar mechanism to that of an EDA complex, and results in the formation of a neutral radical pair [90] (Scheme 2B). Due to the mechanistic similarities, these complexes must be studied in depth to determine whether the reaction proceeds via an EDA or CIP complex [91].

The frequency at which these radical intermediates form the desired products is restricted by the rate of back-electron transfer (BET) [54], which happens at a faster rate than the rate of diffusion, limiting intermolecular reactions. To combat this, leaving groups are often attached to the donor or the acceptor components to force an irreversible transformation (rate constant of *k*_F_) of the radical ion pair, which, when faster than the rate of BET, allows for the reactive processes to occur [92, 93] (Scheme 2C).

EDAs have been rapidly emerging as a new field for research within photochemistry [56, 94–96]. As previously discussed, EDA photochemistry does not require an exogenous photocatalyst, has a propensity for mild reaction conditions, and is a powerful method for formation of C-X bonds [97], due to the radical synthesis pathway. This method has also been described as green; due to mild reaction conditions, environmental benignancy [81, 95, 97] and being transition metal photocatalyst-free [57, 58, 98, 99]. As a result, EDA complex photochemistry can enable the activation of compounds using visible light, even if those compounds do not intrinsically absorb in the visible range [55].

### Utilising Flow Chemistry in Photochemistry

The field of photochemistry has been aided with the advent of flow chemistry. Initially, reactions in continuous flow were performed for scale-up on a larger industrial scale [100–102], however, since the late 1990’s, continuous processes have been continually developed and optimised, and have been viewed as a suitable alternative to performing reactions in batch for synthetic reactions [103–106]. The functionality of flow chemistry for photochemical reactions has been explored in depth [107–109]; Noël describes nine significant advantages of the use of flow for photochemical reactions [65], including increased light penetration [66], higher efficiency of mixing [110, 111] and increased yields [112–114] among other things. Firstly, one key advantage of flow photochemistry over batch photochemical reactions [115] can be readily understood through the basis of the Beer-Lambert [116] Law (Equation 1).

1 $$\text{log}\left(\frac{{I}_{0}}{I}\right)=A=\varepsilon cl$$

**Fig. 2:**
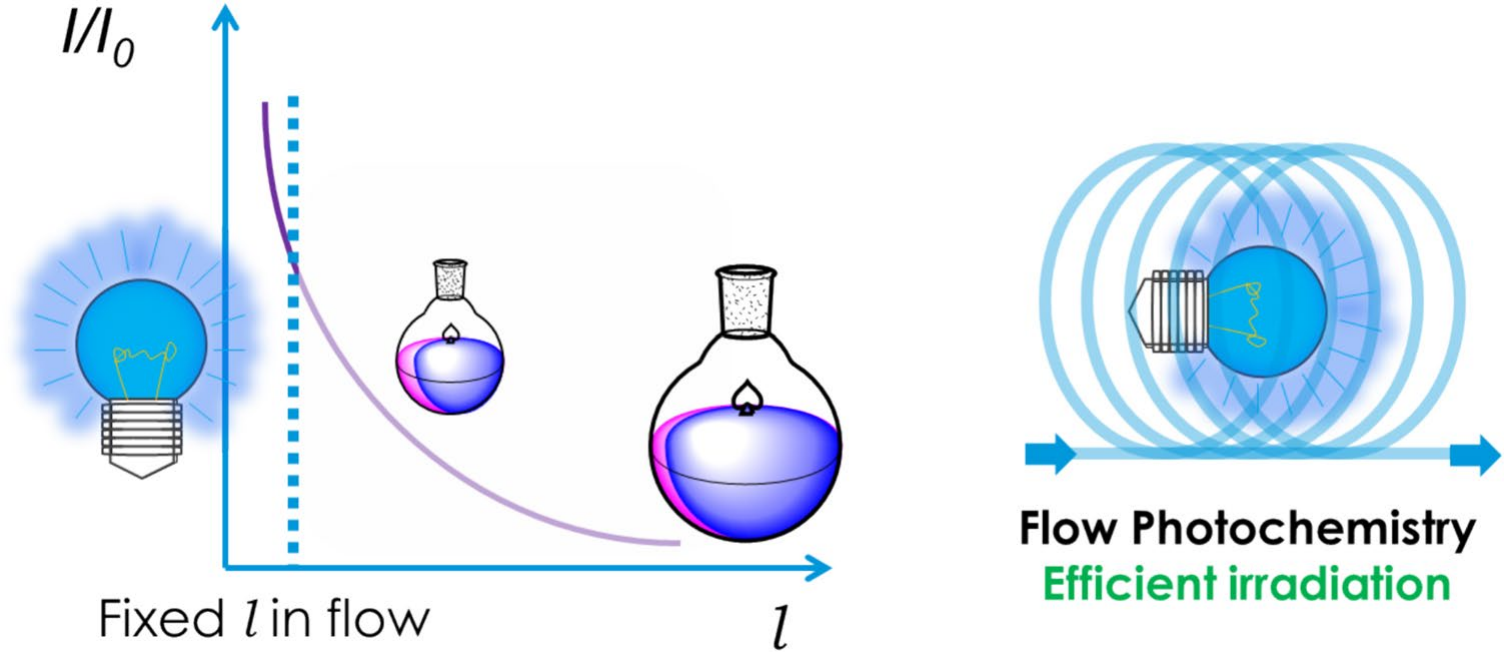
A cartoon demonstrating the contrast between batch and flow when scaling up light-driven reactions, showing the difference between a large batch flask and a photo- flow reactor

From this equation, it can be deduced that if the path length (l) is large, as it would be when scaling reactions up in batch to gram or even kilogram scale using larger reaction vessels (with diameters in centimetres or possibly meters), the absorbance (A) would increase, meaning the final intensity (I) of light in the centre of the reaction vessel would exponentially decrease. The inequality of irradiation leads to both over-irradiation on the edges and under-irradiation in the centre, often leading to the formation of side products and photopolymers [108]. Therefore, even for the use of the greener EDA synthetic technique, the advantages are somewhat negated by the inability to scale-up these reactions to a larger gram-scale in batch. To counter this, developments in the field of flow chemistry have allowed for the thin tubing to be an excellent vessel for photochemical reactions. The thinner path-lengths of the microreactor tubing (diameters less than one millimetre) and small volumes allow for more of the reaction solution to “see” the light, providing for a much more uniform efficient irradiation of the reaction solution [64–66, 108, 109] (Figure 2). This can be displayed graphically in a transmission profile, wherein as the path length increases, the transmission decreases exponentially [116] (Figure 2).

Typical photochemical flow reactors will have tubing, often made of perfluoroalkoxy alkane (PFA) or ethylene tetrafluoroethylene (ETFE), coiled efficiently around a light source, or have a light source surrounding the tubing [117], leading to uniform irradiation. This efficiency of irradiation will not only increase reaction yields on small scale but can be successfully applied to larger volumes of reaction solution to enable scale-up.

Additionally, flow chemistry has been found to have a positive impact on selectivity [118], which can be attributed to increased surface-to-volume ratios within the microtubing, in conjunction with easier and more accurate control of quenching and precisely controlled reaction and residence times [65]. Residence time (t_R_) describes the average amount of time the reaction solution is positioned within the reactor under reaction conditions (i.e. heat or light) and is calculated through Equation 2. The residence time can be changed through the altering of the flow rate of the solution within the apparatus.

2 $$Residence time \left({t}_{R}\right)=\frac{Reactor Volume}{Flow Rate}$$

Noël’s nine reasons [65] also include the ability to complete multistep reaction sequences within the same reaction manifold, immobilisation of photocatalysts to enable ease of recycling and recovering of the photocatalyst, and increased safety. One other additional advantage of flow chemistry over batch reactions is the ability to monitor reactions continuously within the flow manifold itself.

**Fig. 3:**
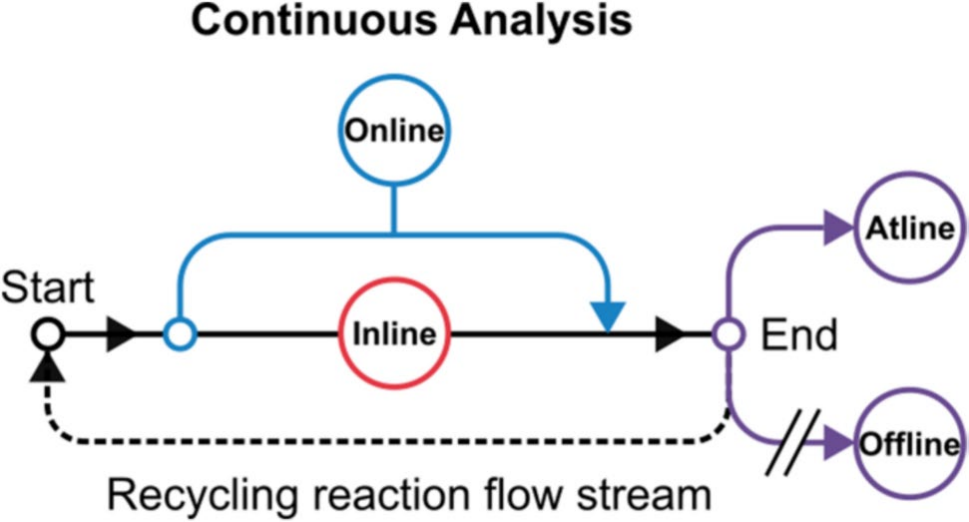
Schematic summarising the definitions for online, inline, atline and offline analysis in relation to a continuous flow system. *Reproduced from Patterson, Wong, Barker and Vilela *[129]* with permission from Springer, Creative Commons Attribution 4.0 International License *[130]

These real-time monitoring techniques and control capabilities, enable immediate detection of products and reactants alongside continuous real-time monitoring of the progress of the reaction, using techniques such as NMR (Nuclear Magnetic Resonance), UV-vis, HPLC (High-Performance Liquid Chromatography) and IR (Infared) spectroscopy [119–123]. Subsequently, inline and online techniques can allow for optimal conditions and high product quality to be discovered and established, reducing the risk of process deviations and waste. Additionally, inline analysis minimizes the need for sampling and offline testing, enhancing both safety and efficiency. These analytical techniques are performed in a range of different approaches with respect to the flow manifold and are commonly described using a qualifying prefix. Despite some initial confusion, several reviews have set out to define the terms of inline, online, offline and atline, culminating in what appears to be an agreed upon consensus [124–129] (Figure 3).

These analytical techniques in relation to continuous flow are described as followed; inline is a continuous process within the flow manifold without any alterations to the reaction stream or changes to process conditions (such as a temperature probe). Online is similar, but does not take place within the reaction stream, which is diverted outside the original operating procedure (examples include continuous NMR monitoring of the reaction solution in a photochemical process where light is not present in the NMR probe). Atline techniques are those where the reaction solution is not fed back into the reaction pathway after analysis (such as a HPLC to purify products), whilst offline is defined as techniques where the samples are removed from the flow manifold manually and not returned (such as Gel permeation chromatography (GPC) analysis) [129, 131]. These techniques can be utilised in a variety of different approaches and have their own merits and drawbacks.

As explored here, flow chemistry offers several advantages over traditional batch processes, including improved reaction efficiency [65, 108–111], better control over reaction conditions [65, 108, 109], and enhanced safety [129]. Continuous flow photochemistry allows for precise control of reaction conditions, leading to increased reaction rates [63, 65] and in some cases, selectivity [63, 118]. A flow manifold can also utilise reaction monitoring techniques to continuously monitor the progress of a reaction in such a manner that is not possible in batch, giving flow a significant advantage via the ability to track the progress of a reaction that is underway [119–123]. These significant benefits therefore can be applied to studies of EDA photochemical reactions, which could lead to some interesting innovations and will expand the field of photochemistry in flow to reactions not involving photocatalysts. This review includes research work published up to and including January 2025.

## Using flow as a scale-up technique

In this section, we explore research that has utilised flow to scale-up certain photochemical reactions that proceed via EDA complexes. As discussed in Section "EDA photochemistry", the scale-up of photochemical reactions is more viable in flow, mainly due to the smaller path length of the thin tubing, allowing for a more uniform irradiation of the reaction solution.

**Scheme 3:**
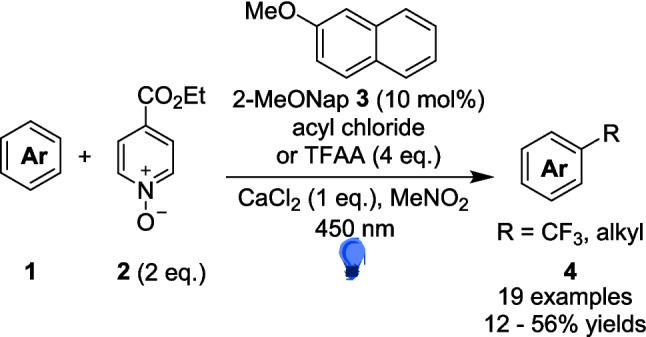
EDA complex catalysis for radical trifluoromethylation and alkylation [132]

**Scheme 4:**
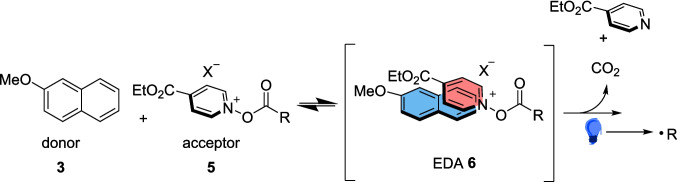
EDA complex formation through a π-stacking interaction for catalysis for radical trifluoromethylation and alkylation, leading to the formation of the radical [132]

In 2020, Stephenson developed a method for trifluoromethylation and alkylation of aromatics exploiting an EDA (Scheme 3) complex formed from 2-methoxynaphthalene 3 and acylated ethyl isonicotinate N-oxide 5 (Scheme 4) [132]. In this reaction, 2 reacts with either acyl chloride or TFAA to form 5. The reaction between a donor aromatic Substrate 3 and the acceptor 5 generates an EDA complex 6 through a π-stacking interaction [133], which acts as the radical precursor for this general reaction (Scheme 4).

**Scheme 5:**
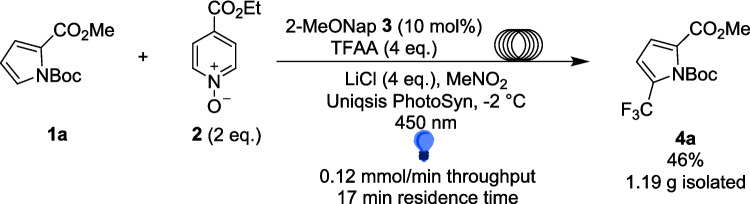
Scale-up of the EDA complex catalysis for radical trifluoromethylation and alkylation in flow [132]

The use of extended π-systems in donors Such as naphthalene, Supplied more absorbing EDA complexes, while an electron-donating substituent, such as methoxy in 3, was able to increase the reactivity of the EDA. Absorbance of the CT band also increased with both the polarity of the solvent and concentration. On 0.2 mmol scale (batch), the trifluoromethylation reactions produced isolated yields of 14-56% for a range of aromatic Substrates 1, including pyrroles, indoles and benzene derivatives. This led to the exploration of the scale-up of the reaction utilising a plug-flow reactor (Scheme 5). The initial efforts failed, due to calcium chloride within the reaction becoming opaque and gelatinous, which clogs a flow reactor set up. Calcium chloride was subsequently exchanged for lithium chloride, which provided a heterogeneous, free-flowing reaction mixture while additionally increasing the absorptivity of the EDA complex. The reaction with methyl N-Boc pyrrole-2-carboxylate 1a originally produced a 39% isolated yield of 4a in the 0.2 mmol scale reactions, but after utilising a Uniqsis Photosyn plug-flow reactor [134], the authors were able to scale-up the reaction to 2 mmol and produce 1.19 g or a 46% yield of the desired product 4a, a 10-fold increase in scale compared to the same reaction in batch and producing a higher yield (vs. 0.0228 g, 39% yield 4a in batch). It should be noted, however, that since this work was focussed mainly on method development on small scale in batch, only one model reaction (Scheme 3) was investigated in flow (Table 1)

**Table 1:** Tabulated results for the formation of product 4a in both batch and flow [132].

Flow/Batch	Reaction Time	Scale	%age Yield
Batch	1 h	0.2 mmol	39%
Flow	17 min	2 mmol	46%

**Scheme 6:**
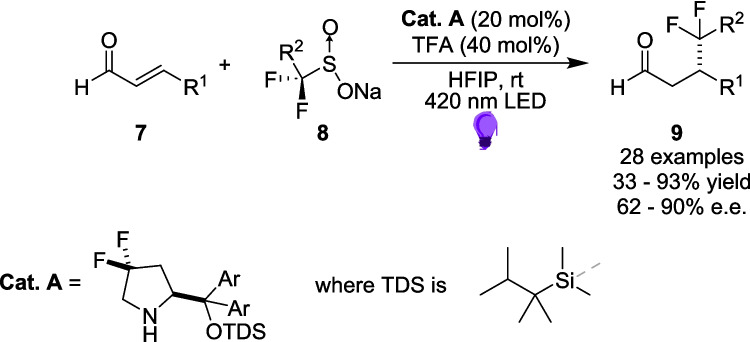
Asymmetric transformation for the diflouroalkylation of α, β-unsaturated aldehydes, where Cat. A is an organocatalyst [135]

Two years later, Alemán *et al.* were able to show a greater range in the application and adaptability of flow reactions when conducting their investigations into sulfinates as a source of alkyl radicals for use in the enantiocontrolled β-functionalisation of enals 7 [135]. Their search for a diflouromethylation technique that does not include ozone-depleting substances [136] led them to a radical pathway involving fluorinated Sulfonyl radical precursors 8 (Scheme 6). This approach requires an organocatalyst **Cat. A** and was performed initially on small scale in batch.

**Scheme 7:**
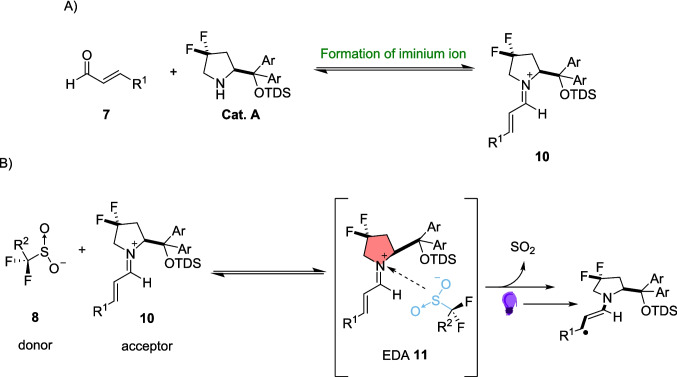
The key steps in creating the EDA radical precursor **A**) the formation of the iminium ion between enal 7 and the organocatalyst Cat. A and **B**) formation of the EDA and Subsequent radical between the acceptor iminium ion 10 and the donor Sulfinate 8 [135]

These Sulfinates 8 have increasingly been used as reagents in organocatalytic reactions due to their high stability, commercial availability and ease of handling [137, 138]. The reaction of enal 7 with organocatalyst Cat. A forms the iminium ion 10 (Scheme 7A), before the formation of the EDA complex 11 (Scheme 7B), upon reaction of the Sulfinate 8 with the iminium ion 10.

However, following the review by Stephenson [54], and the research by Kochi [89, 93], this type of interaction between charged substances is now better described as a CIP. CIPs have similar chemistry and photoactivity as EDAs but instead result in the formation of a pair of neutral radicals after photoinduction, rather than a radical ion pair produced by an EDA (Scheme 2B).

**Scheme 8:**
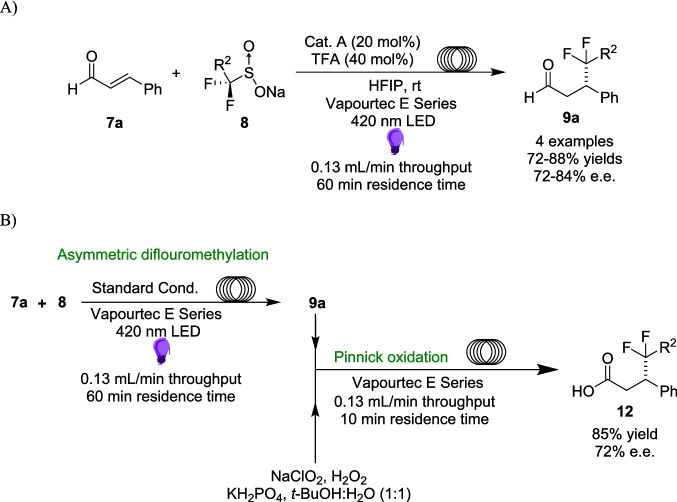
Photo-flow setup of **A**) the asymmetric diflouromethylation and **B**) the two step approach including a further oxidation step to produce a synthetically relevant product [135].

Regardless of the mechanism, the reaction, on a 0.2 mmol scale, had a Substrate scope of 10 enals, 8 diflouroalkyls Sulfinates, 6 diflouroaryls Sulfinates and 4 alkyl Sulfinates, with yields ranging from 33−93%. Crucially, these reactions were also enantioselective, with *e.r.*s ranging between 81:19 to 95:5. Transferring four examples of this reaction to a commercial *Vapourtec E Series* photoflow reactor[139] (Scheme 8A), Alemán was able to closely Match these results, with yields between 72−88% and *e.r.*s between 91:9 to 96:4 while increasing the scale to 1 mmol. However, the reaction proved to be much more efficient in flow, with a time frame reduced from 5 hours to a single hour, resulting in a 30-fold increase in reaction throughput.

Following these promising results, an additional oxidation step was added within the flow Manifold, providing the final product, a bioactive diflouroalkyl derivate of a carboxylic acid 12 (Scheme 8B). Product 12 is a key synthetic step for the selective inhibition of Ubiquitin-specific-processing protease 7 (USP7) [140, 141], which is prone to cancerous mutations. Another synthetically significant advancement was that this step has now been reduced from a six-step synthesis to just one continuous flow system, producing the product 12 on a 1 mmol scale.

**Scheme 9:**
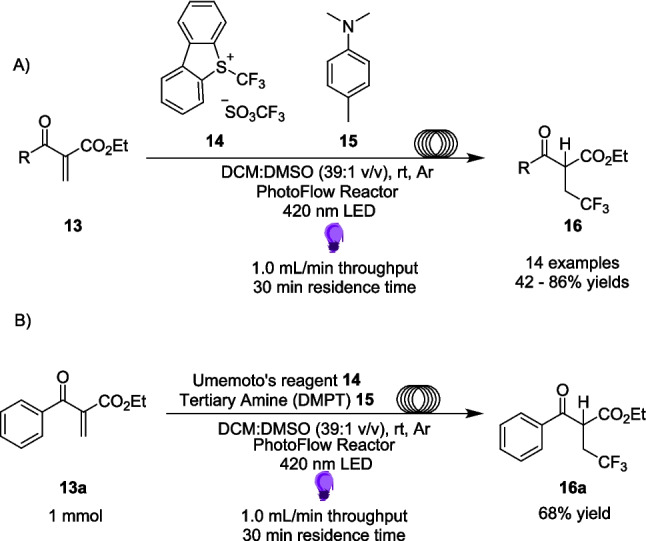
Hyrotriflouromethylation of β-keto esters performed solely in flow with **A**) the general procedure performed at 0.2 mmol scale and **B**) the procedure of the reaction which was scaled up to 1 mmol [142]

Further research into flouromethylations *via* EDA complexes in flow was performed by De Olivera and Amarante in 2020 [142]. Building on the work of Li [143] and Xu [144], they proposed an approach for the hydrotriflouromethylation at the β carbonyl position of unsaturated β-keto esters **13**. Uniquely, this approach was performed on 0.2 mmol scale solely in flow (Scheme 9A), in order to exploit the benefits of flow, including greater efficiency, shorter reaction times and high scalability. The higher efficiency irradiation combined with the ratio of the tertiary amine 15 to the Umemoto’s reagent 14 led to the hypothesis that the yield will be greatly increased in comparison to previous attempts. Importantly, this does not include a transition metal catalyst [143, 144].

**Scheme 10:**
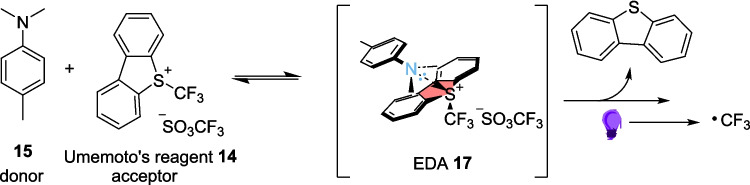
Formation of the EDA 17 between the donor DMPT 15 and Umemoto’s Reagent 14 [142]

The EDA 17 is formed through the combination of the donor dimethylpropiothetin (DMPT) tertiary amine 15 and acceptor Umemoto’s reagent 14 (Scheme 10). The reaction scope of hydrotriflouromethylation of dicarbonyls included 14 different compounds, with yields of the final product ranging between 30-86%. Overall, the aryl-substituted derivatives (R=Ar in 13) were well tolerated. The attempted scale-up of the reaction was performed on 1 mmol scale, and resulted in a yield of 68%, a slight decrease from the 0.2 mmol scale of the same reaction which produced a yield of 74%. This lone example provides an interesting insight into how yields can change when scaling up purely within flow, and this could be a fascinating path to explore in the Future. Further reactions on 16a were performed, however unlike Alemán’s example discussed in Scheme 8B [135], these were performed by removing the product from flow and performing further reactions in batch.

**Scheme 11:**
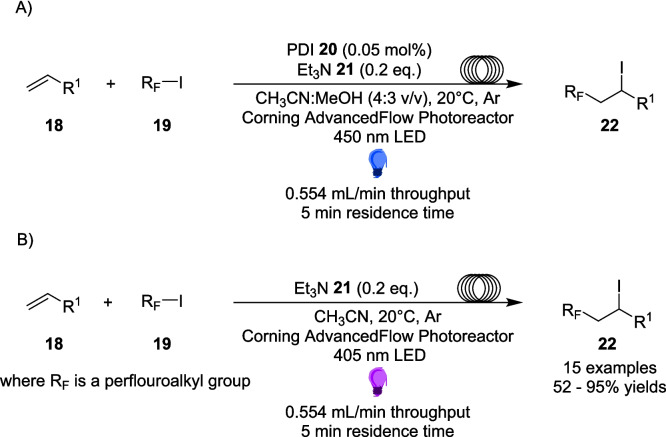
Outline of the iodoperfluoroalkylation of alkenes performed in flow, **A**) requires a PDI photosensitiser while **B**) does not, and proceeds through an EDA pathway [145]

Additional to Alemán, De Oliviera and Amarante, further work into the important field of fluorination reactions is explored in Kappe’s 2019 work, which presents two efficient, scalable methods for iodoperfluoroalkylation of alkenes using visible light under continuous flow conditions [145]. The iodoperflouroalkylation of olefins has been used as a strategy to unlock fluorinated compounds commonly used in agricultural and medicinal chemistry [146], while renewed interest in synthetic organic photochemistry has led to the development of a number of new iodoperfluoroalkylation reactions [146]. However, these reactions have employed expensive transition metal (Ru, Ir or Cu) catalysts [147–149], high loading organic dyes [150, 151] or require the addition of several additives [152, 153]. In an effort to seek out a metal-free photocatalytic approach, the group have previously discovered an efficient method, driven by the addition of a low-loading organic photosensitiser, where yields of 93-98% were originally reported at a 0.1 mmol scale, however this dropped to 57% at a 1 mmol scale [154]. This current work had originally set out to explore how to adapt this reaction for continuous flow conditions (Scheme 11A), and improve yields when performing the reaction at larger scales.

**Scheme 12:**
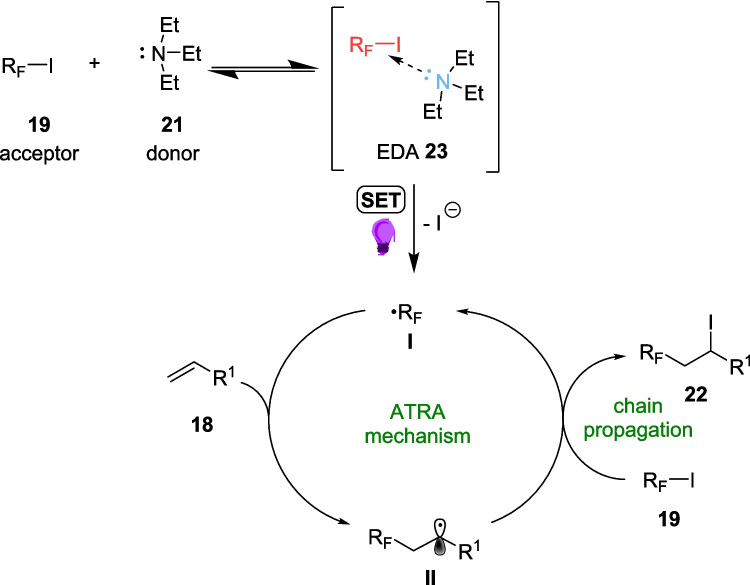
Mechanism for the iodoperfluoroalkylation of alkenes via an EDA formed between perfluoroalkyl iodine 18 and triethylamine 20 [145, 154]

However, during the optimisation process, an alternative approach was discovered, one that did not require perylene diimide (PDI) 20 as an organic photosensitiser, and performed well at shorter wavelengths (Scheme 11B). The inclusion of triethylamine 21, originally utilised as a soluble electron donor, now acts as a doner within an EDA complex, formed between the perfluoroalkyl iodine 18 and triethylamine 21. Once excited by the lower wavelength light, the radical formed then follows an atom transfer radical addition, or ATRA mechanism (Scheme 12).

**Scheme 13:**
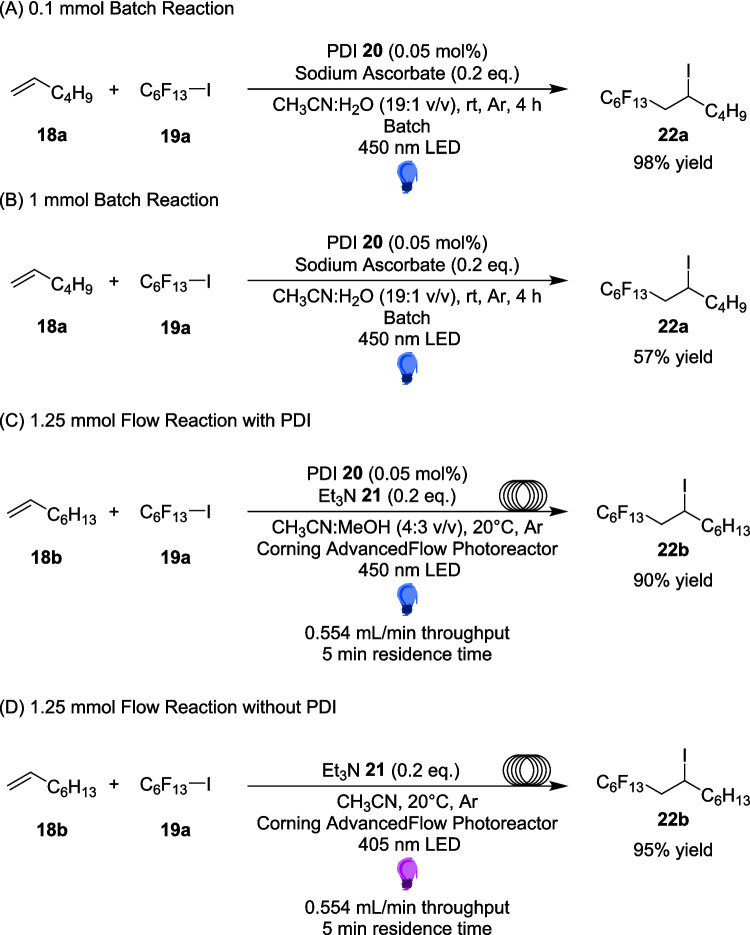
Comparison of both the PDI-assisted and EDA pathway reaction yields for the iodoperfluoroalkylation of alkenes 18a and 18b in both flow and batch [145, 154]

This catalyst-free approach was then investigated Further in flow, after 22b was synthesised at a higher yield, 95%, in the photocatalyst-free approach (Scheme 13D) than both the batch, where 22a was the product scaled up to 1 mmol scale at a yield of 57% (Scheme 13B), and flow approaches which utilise PDI 20 (Scheme 13C). The Successful scale up of the reaction, in both the photosensitiser-assisted and EDA pathway, demonstrates the ease of scalability of flow. The reaction also was able to be completed within the 5-minute residence time in flow, a 48-fold increase in efficiency to the 4-hour batch reaction time, allowing for large scale synthesis to be performed at a much faster rate.

**Scheme 14:**
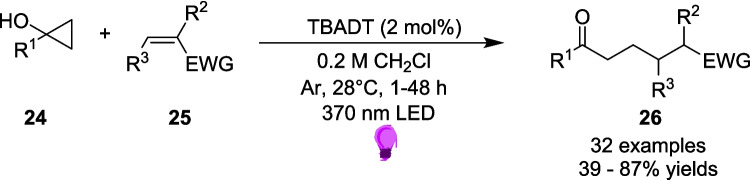
Outline of the TBADT-assisted ring-opening reaction of cyclopropanols using electrophilic alkenes [155]

At the end of 2023, Ošeka *et al.* investigated the ring-opening reactions of cyclopropanols 24 and Subsequent Giese additions onto electrophilic alkenes 25 [155]. Their approach uses a tetrabutylammonium decatungstate (TBADT) photocatalyst (Scheme 14). The application of TBADT as a photocatalyst undergoing a SET oxidation pathway was first explored by Fagnoli and Ravelli [156, 157].

**Scheme 15:**
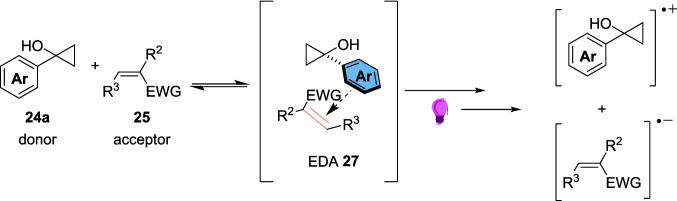
Formation of the EDA between an aryl-derivated-cyclopropanol 24a and an electrophilic alkene 25 [155]

**Scheme 16:**
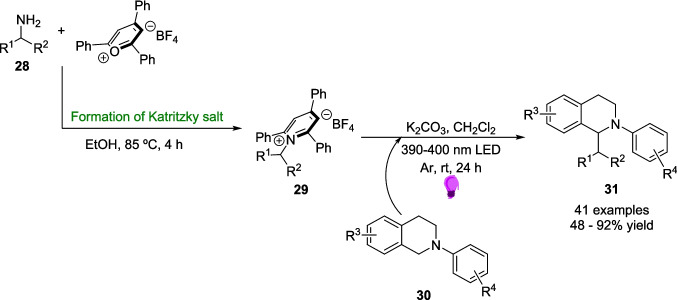
Outline of the light-driven α-alkylation of *N-*Aryl tetrahydroisoquinolines [158]

The researchers found that with certain aryl-derivative- cyclopropanols 24a, the reaction still proceeds without the TBADT catalyst, due to the formation of an EDA between the donor aryl-derivative -cyclopropanol 24a and acceptor alkene 25 (Scheme 12). However, this reaction does not proceed as quickly as the reaction with TBADT as a photocatalyst. In 3 hours, the photocatalysed reaction as shown in Scheme 11 proceeds to an 84% yield of 26, however the reaction without TBADT only reaches a yield of 25% in the same time frame. A yield of 77% was only achieved after 24 h of irradiation Scheme 15 and 16.

There were only two model examples of this reaction investigated in flow, using only the TBADT photocatalyst pathway. This would have been an excellent opportunity to investigate approaches in flow with and without the photocatalyst to compare results, find the most efficient approach to this synthetic pathway, and whether the EDA pathway would become more efficient in flow. An investigation following this process would provide an excellent basis for future research.

## Using Flow to impact efficiency on a smaller scale?

Flow chemistry is not only utilised as a technique to scale-up photochemical reactions but can also be used to increase efficiency by enhancing reaction kinetics on both the smaller and larger scale reactions. As we explore in this section, a positive impact on efficiency can be observed even on smaller scale reactions as the effects of the Beer-Lambert law still hold (Section "Utilising Flow Chemistry in Photochemistry"). This section also presents an example where flow was primarily employed to improve reaction kinetics and, consequently, reaction efficiency, rather than employing flow solely for scaling up. While the researchers do ultimately scale up to gram scale, their primary focus is not on this aspect. The approach of utilizing the higher reaction efficiency of flow was employed by Zhang in two different visible light driven EDA approaches.

In 2020, the W. Zhang group discovered an EDA approach for the α-alkylation of *N-*aryl tetrahydroisoquinolines 30 [158]. These products have been previously synthesised through the oxidative Strecker reaction [159, 160] or metal-catalysed cross-coupling pathways [161, 162] but the work of J. Zhang demonstrated an EDA pathway for the α-allylation [163] that inspired W. Zhang’s work.

**Scheme 17:**
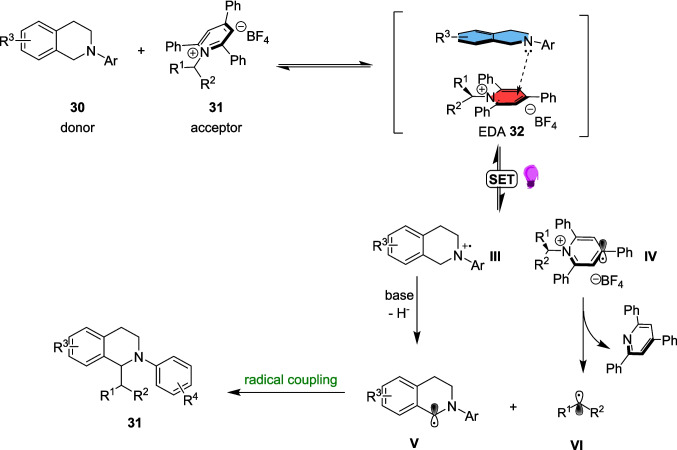
Outline of the mechanism for the light-driven α-alkylation of *N-*aryl tetrahydroisoquinolines 30, involving the formation of EDA 32 between a *N-*Aryl tetrahydroisoquinoline 30 and a Katritsky salt 31 [158]

This approach (Scheme 13) involves the formation of an EDA between a N-aryl tetrahydroisoquinoline 30 and a Katritzky salt 29, the latter of which are readily prepared from their respective amine 28. The EDA 32 is then excited under 390-400 nm light-emitting diode (LED) light and forms alkyl radical VI and α-amino radical V which then combine to form the desired product 31 (Scheme 17).

**Scheme 18:**
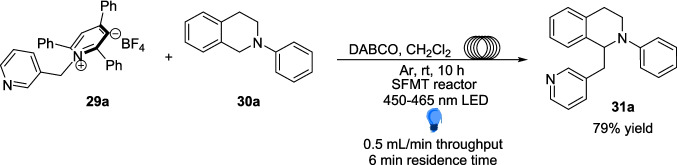
A model reaction for the α-alkylation of *N-*aryl tetrahydroisoquinolines 30 in an SFMT reactor [158]

This reaction was also studied in flow with slight changes to the original approach (Scheme 18). Here, the K_2_CO_3_ salt is exchanged for 1,4-diazobicyclo[2.2.2]octane (DABCO) and the 390-400 nm LEDs exchanged for 450-465 nm lights. Although not explicitly discussed by the authors, the change of base is very likely due to the insolubility of K_2_CO_3_ in DCM, which may cause blockages within a flow set-up. However, it is not clear why the lights were altered for the flow set up, especially since the optimisation reaction in blue light (62% yield) performed worse than the standard conditions explored above (72% yield) in batch optimisation. Nevertheless, the reaction within a stop-flow microtubing (SFMT) reactor was performed at the same 0.2 mmol scale as in batch. This approach was justified to determine the synthetic applicability of this EDA pathway and demonstrate a different practical approach to Light-mediated catalyst-free reactions. The desired product 31a was synthesised in 79% yield within 10 hours, as compared to the same reaction in batch which produced 31a in a yield of 72% after 24 hours (Table 2).

**Table 2:** Tabulated results for the formation of product 31a in both batch and flow [158].

Flow/Batch	Reaction time	Scale	%age Yield
Batch	24 h	0.2 mmol	72%
Flow	10 h	0.2 mmol	79%

This SFMT approach was only explored on one example but was still able to demonstrate the increase in efficiency that a continuous flow Manifold can provide for photochemical reactions containing EDA complexes. Curiously, the group opted to scale-up the reaction to gram scale, or 4 mmol, in batch rather than in flow, producing a variant of product 31 at a yield of 84%, compared to 92% on the smaller 0.2 mmol batch scale.

**Scheme 19:**
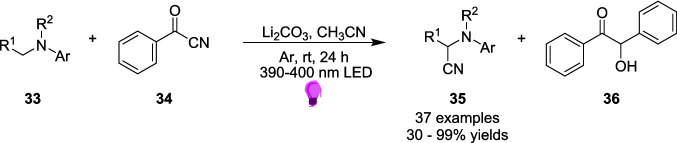
Outline of the Light-driven photocatalyst-free cyanation of arylamines 33 to produce α-amino nitriles 35 [164]

Zhang and Wang’s later work in 2020 introduced an approach for the cyanation of arylamines 33 to produce α-amino nitriles 35 via an EDA complex pathway (Scheme 19) [164]. This type of reaction has previously only been performed via the Strecker reaction [165, 166] or via a cross-dehydrogenative coupling (CDC)-type reaction [167, 168] until a visible-light induced photocatalyst method was introduced in 2011 [169, 170]. Zhang and Wang’s approach seeks to remove the photocatalyst and develop a cyanation technique involving the use of EDA complexes. This approach utilised benzoyl cyanide 34 as the source of cyanide and a range of tetrahydroisoquinolines (THIQs) and N-aryl alkylamines as the arylamine 33 sources (Scheme 19).

**Scheme 20:**
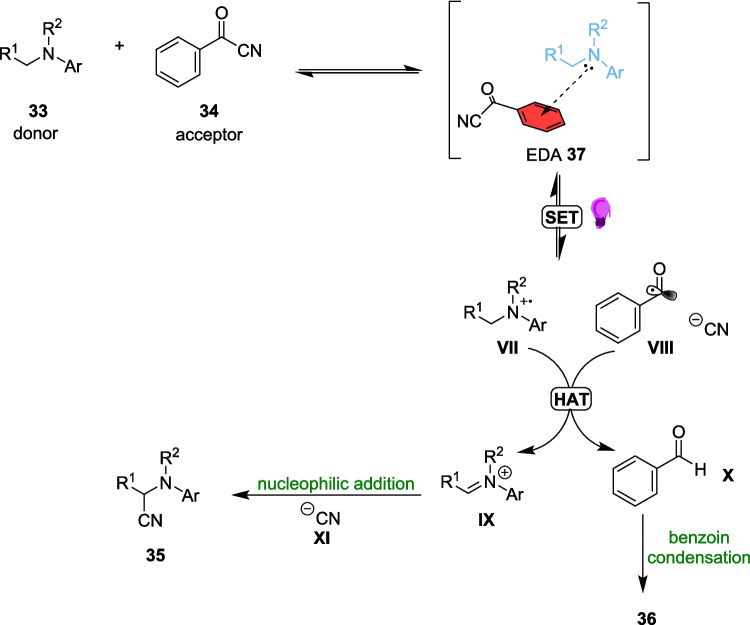
Outline of the mechanism for the Light-driven cyanation of arylamines 33 to produce α-amino nitriles, involving the formation of EDA 37 between an aryl amine 33 and benzoyl cyanide 34. This mechanism also involves the formation of by-product 36 [164]

Reactants 33 and 34 combine to form an EDA 37 and, after irradiation with a 390-400 nm Light, the reaction proceeds through a radical pathway to form product 35 and by-product 36 (Scheme 20). By-product 36 is produced via the benzoin condensation reaction, one of the oldest known reactions in organic chemistry [171], and is produced independently of the final product and so the formation of 36 does not impact the yields of 35.

**Scheme 21:**
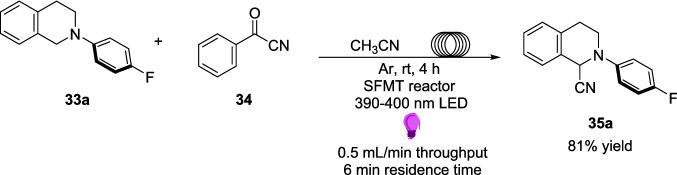
A model reaction for the production of an α-amino nitrile 35a from 2-(4-flourophenyl)−1,2,3,4-tetrahydroisoquinoline 33a and benzoyl cyanide 34 [164]

Much like with Zhang’s previous work, a SFMT reactor was utilised to demonstrate the reaction’s Suitability in flow. Again, the reaction was altered slightly to remove the Lithium carbonate, thus removing a solid that could cause blockage problems in flow. In the batch optimisation studies at 0.1 mmol, this alteration led to a yield of 65% after 48 hours, as compared to the initial approach (Scheme 19) which led to a yield of 98% after 24 hours. The reaction was again performed at the same 0.2 mmol scale as the batch substrate scope (Scheme 21), with the product 35a produced in 81% after 4 hours, compared to 97% after 24 hours in the respective batch reaction. Despite the drop in yield, this still demonstrates the positive utilisation of flow for this type of EDA reaction, since the product is still formed in a good yield within a much smaller timeframe (4 vs. 24 h) (Table 3).

**Table 3:** Tabulated results for the formation of product 35a in both batch and flow [164].

Flow/Batch	Reaction time	Scale	%age Yield
Batch	24 h	0.2 mmol	97%
Flow	4 h	0.2 mmol	81%

As with Zhang’s previous work, this model approach was explored on this singular example, as well as scale-up attempts being attempted solely in batch conditions, where an example of product **35** was synthesised on an 8 mmol scale, leading to a yield of 80%, or 1.03 g after 36 hours. The Identical reaction on the 0.2 mmol scale produced a yield of 81% after 24 hours. As presented in the examples in the previous section, a continuous flow manifold provides an excellent opportunity to scale-up photochemical reactions without witnessing a significant drop in yield as well as providing a faster reaction time. Therefore, it would be of great interest to see these reactions scaled up in flow, not only to compare to the small-scale flow set ups, but also to compare the reaction efficiency to the batch scale-up attempts.

**Scheme 22:**
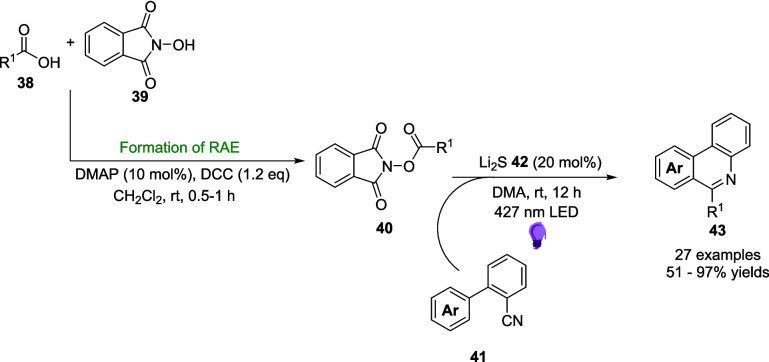
Outline of the alkylation of isocyanides 41 using RAEs 40. This scheme also involves the synthesis of the RAE [164, 172]

In 2022, Wang described a broad and versatile procedure for decarboxylative alkylations without the use of a photocatalyst (Scheme 22) [172]. Sulfur-based photosensitisers have been used in this type of Light-induced transformation since at least 2009 [173, 174] due to the excellent radical properties, and electron transfer ability [175] of sulfur-containing compounds as well as sulfur’s ability to hold a valance state anywhere between −2 and 6 [176]. Despite these clear advantages, sulfur-containing photosensitisers are both expensive and complex to synthesise and thus an inexpensive pathway is much more desirable. Shang and Fu reported an EDA approach for decarboxylative alkylations without a photosensitiser [177]. Combining this approach with those exploiting the properties of sulfur, Wang describes an EDA pathway involving sulfide anions (Scheme 20).

**Scheme 23:**
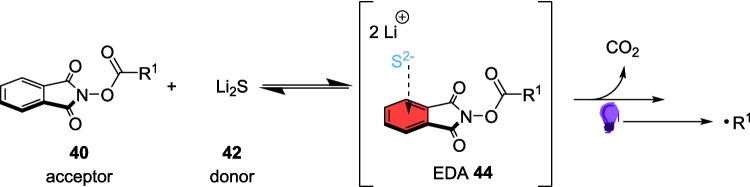
Formation of the EDA 44 between the RAE 40 and lithium Sulphide 42 [172]

This reaction utilises redox active esters (RAEs) 40, synthesised through the addition of the corresponding carboxylic acid 38 and N-hydroxyphthalimide 39, as the source of the alkyl radical and an isocyanide 41 as the Substrate. The EDA is generated between the RAE 40 and lithium Sulphide 42 to generate the alkyl radicals (Scheme 23), which then proceed to react with isocyanide 41 to form the product 43 (Scheme 22).

The reaction was also investigated in a one-pot approach, synthesising the RAE 40 in situ then performing the alkylation of isocyanide 41. The one-pot approach performed admirably to produce an 86% yield of 43a, compared to 92% from the two-step approach where 40 is isolated first.

**Scheme 24:**
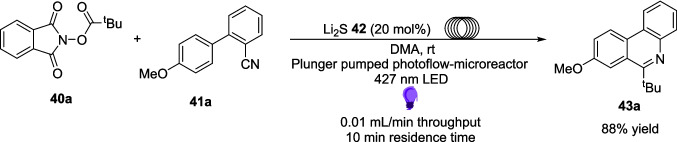
A model reaction for the alkylation of an isocyanide 41a using a RAE 40a and lithium Sulphide 42 in flow. This reaction produces product 43a in a high yield after 10 minutes, compared to the 12 hours in the standard batch conditions [172]

The reaction was also studied using continuous flow (Scheme 24), as a technique to improve the efficiency of the reaction. To be able to adapt the reaction for flow, the reaction was carried out on a 6 mmol scale compared to the batch reactions at 0.2 mmol. In batch, the equivalent scale-up experiment produced 43a in an 85% yield after 12 hours, while when translated to flow, this reaction achieved a Marginally better yield of 88% after a reaction time of just 10 minutes, an astounding 72-fold increase in efficiency (Table 4).

**Table 4:** Tabulated results for the formation of product 43a in both batch and flow [172].

Flow/Batch	Reaction time	Scale	%age Yield
Batch	12 h	6 mmol	85%
Flow	10 min	6 mmol	88%

**Scheme 25:**
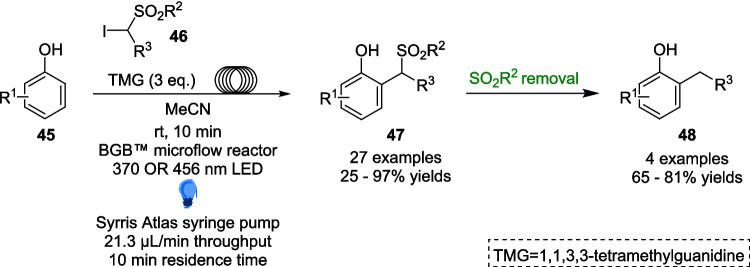
Outline of the alkylation of phenols 45 within microfluidic conditions [178]

Another further example can be explored in the work of Filippini and Dell’Amico in their 2022 work, which presents a novel method for the direct alkylation of phenols using visible light within a microfluidic flow setup (Scheme 25) [178].

Phenol derivatives are widely prevalent in natural products, pharmaceuticals, and biologically active functional materials [179–182] and thus a direct and selective C-H functionalisation technique would be of considerable interest. As with previous examples, progress in this discipline has been limited to reactions catalysed by transition-metal or organic based catalysts which are able accomplish ortho (*o*)- or para (*p*)- selectivity and require high temperatures and toxic catalysts [183–185]. Melchiorre has previously explored a sustainable alternative method to the direct C-H functionalisation of phenols via homolytic aromatic substitution (HAS) and a radical pathway [186], utilising phenolate anions to drive the formation of perfluoroalkyl radicals under simple and mild conditions, albeit still requiring pre-functionalised Substates, and a reaction time of 16 hours.

**Scheme 26:**
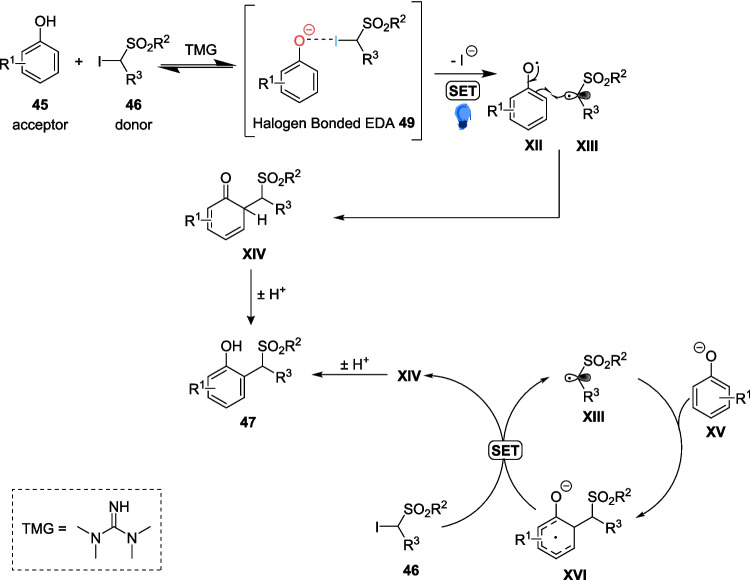
Proposed mechanism for the photoalkylation of phenols 45, including the formation of halogen bonded EDA 49 [178]

This recent work employs the use of *α*-iodosulfones 46 under basic conditions, using 1,1,3,3-tetramethylguanidine (TMG) as a strong, non-nucleophilic base[187]. When these α-iodosulfones 46 are combined with the phenolate anion, forms a halogen bonded EDA complex 49. Upon Light radiation, a radical mediated HAS process leads to the formation of the alkylated phenol 47 (Scheme 23), which then can be desulfonylated according to a simple reductive cleavage reaction from literature [188] to form product 48 (Scheme 26).

**Scheme 27:**
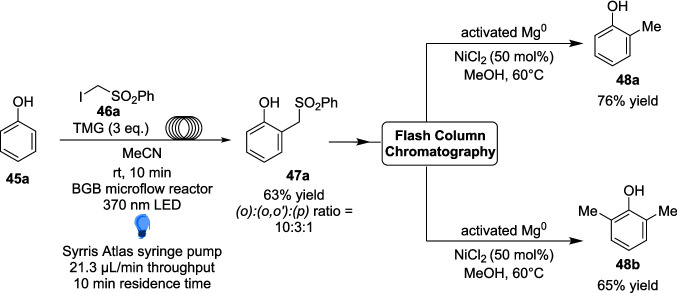
Example of the two-step methylation process of an unsubstituted phenol 45a [178]

The scope of the reaction included 27 different Substrates, including biologically relevant phenols Such as tyrosine, paracetamol and methyl salicylate, with yields ranging from 25 to 97%. However, most importantly, the reaction bore results of up to >20:1 regioselctivity in favour of the (*o*)- product, a vast contrast to the complex mixture of regioisomers produced under more classical methylation reactions [189]. The two-step methylation process (Scheme 27) facilitates the targeted production of specific methylated regioisomers, eliminating the need for any separation through high-performance liquid chromatography (HPLC). Additionally, the reaction time of just 10 minutes within the microflow system the reaction can proceed much faster than any previous attempts at the methylation of phenols, and a 95-fold increase in efficiency as compared to Melchiorre’s previous catalyst-free work.

**Scheme 28:**
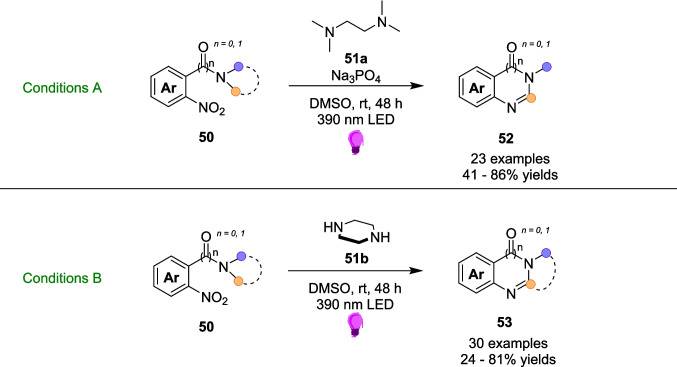
Outline of the visible-light driven synthesis of *N*-heterocycles 52 and 53 from nitroarenes 50 and amine 51, either 51a or 51b [190]

**Scheme 29:**
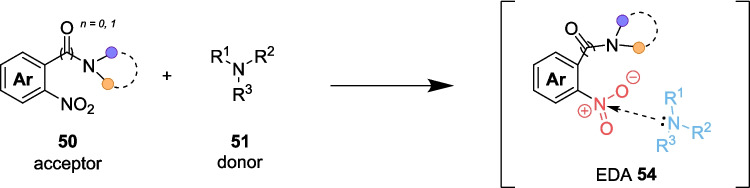
Formation of EDA 54, from the combination of the acceptor nitroarenes 50 and the donor amine 51 [190]

In 2024, the Jin group presented a one-step method for the reduction followed by cyclisation of nitroarenes (Scheme 28) [190]. These *N*-functionalised azahetereocyclic compounds are typical examples of pharmaceutical and biomolecular frameworks, and are found in a large quantity of FDA-approved and marketed drugs [191, 192]. A thermal based intramolecular cyclisation reaction has previously been the principal approach; however, these approaches are performed under harsh conditions and use metal-based catalysts [193–195]. Instead, the Jin group sought to build on the work of Li [196] and Yan [197], by combining the electron-deficient nature of nitroarenes 50 and electron-rich amines 51 (either TMEDA 51a or piperazine 51b) to form an EDA complex 54, thus initiating a reduction process for the synthesis of *N*-heterocycles 52 and 53 (Scheme 29). The reaction using TMEDA, Conditions A, requires a Na_3_PO_4_ base to promote the formation of EDA 54. However, this is not required for Conditions B (Scheme 29).

When the EDA 54 is formed through the combination of the acceptor nitroarenes 50 and the donor amine 51 (Scheme 29), it is irradiated with 390nm Light and undergoes SET and HAT steps to produce the N-heterocycle product 53, although the mechanism and radical formation is not fully known. The reaction conditions are mild and simple, and can be performed as a one-pot transformation.

**Scheme 30:**
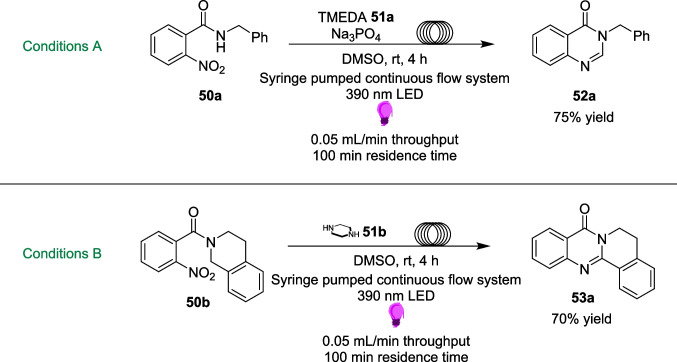
The model reaction of the visible-light driven synthesis of *N*-heterocycles 52a and 53a using TMEDA 51a or piperazine 51b and nitroarenes 50a and 50b [190]

To outline the ease of this one-pot transformation, the reaction was transferred to a flow manifold (Scheme 30), utilising a syringe pump to flow the reaction solution around the light source [190]. This reaction was tested on 4 examples from the Substate scope, and performed on a 1 mmol scale, compared to the original batch scale of 0.3 mmol. Again, this was performed at a larger scale to not only investigate the benefits of scale-up in flow, especially since a larger scale-up was investigated solely in batch, but also to demonstrate the effects of flow on the efficiency of a reaction. Thus, both Conditions A and Conditions B were tested in flow and compared to the corresponding reaction in the batch conditions (Scheme 24). For Conditions A, a yield of 86% for product 52a was achieved in 48 hours under batch conditions. However, in the larger scale flow conditions, the reaction was performed for only 4 hours and achieved a yield of 75%. Similarly, product 53a was achieved in 75% yield in the batch conditions within the same 48-hour timeframe. Within the 4-hour scaled up flow reaction, a yield of 70% was achieved for product 53a. For both these examples, there remains a slight decrease in yield, however the significant reduction in reaction time is a Substantial advantage. The reaction to form 52a was also scaled up to 5 mmol scale in batch (60% vs. 86% 5 mmol vs. 0.3 mmol scale, both 48 h), where the scale-up in flow compares more favourably both in yield and reaction time (75%, 4 h), albeit on a different scale of 1 mmol. It is unclear why different scales were chosen for batch and flow scale-up, which precludes a direct comparison (Tables 5 and 6).

**Table 5:** Tabulated results for the formation of product 52a (*Conditions A*) in both batch and flow [190]

Flow/Batch	Reaction time	Scale	%age Yield
Batch	48 h	1 mmol	86%
Flow	4 h	1 mmol	75%

**Table 6:** Tabulated results for the formation of product 53a *(Conditions B)* in both batch and flow [190].

Flow/Batch	Reaction time	Scale	%age Yield
Batch	48 h	1 mmol	75%
Flow	4 h	1 mmol	70%

## Exploring other merits and drawbacks of flow

In the discussion so far, the benefits of flow with regards to both scale-up and efficiency have been explored. This generally paints continuous flow as an alternative reactor type to explore photochemical reactions involving EDAs. However, due to the nature of flow chemical reactors, there are unconventional uses which can be exploited as well as some inconvenient drawbacks.

**Scheme 31:**
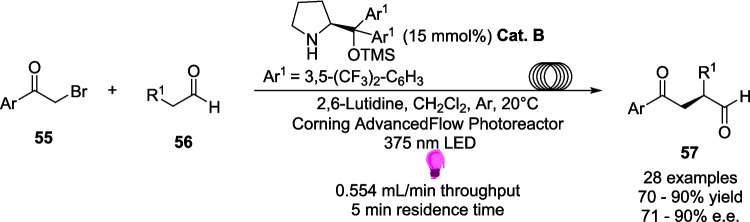
Outline of the asymmetric α-alkylation of aldehydes performed in continuous flow where **Cat. B** is an organocatalyst [198]

In 2023, Kappe and Ötvös set out to explore the enantioselective α-alkylation of aldehydes within continuous flow (Scheme 25) [198]. The visible light-driven α-alkylation of aldehydes has previously been explored by Nicewicz and MacMillan, who discovered an approach using a combination of photoredox and organocatalysts [43], before Melchiorre discovered an EDA pathway without an external photocatalyst [199]. In this research, Kappe and Ötvös modified and adapted Melchiorre’s methodology for continuous flow (Scheme 31).

**Scheme 32:**
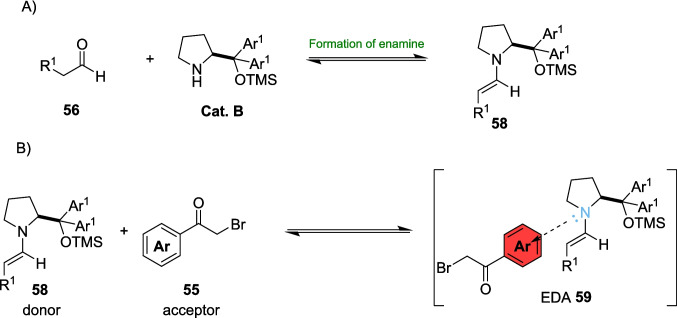
The key steps in creating the EDA radical precursor for α-alkylation of aldehydes *A)* the formation of the enamine 58 between aldehyde 56 and the organocatalyst **Cat. B** and *B)* formation of the EDA 59 between the donor enamine 58 and the acceptor alkyl bromide 55 [135, 198, 199]

This approach utilises a pyrrolidine derivative **Cat. B** primarily as an organocatalyst, which also acts as a precursor to the acceptor for the EDA in a similar way to the EDA formation discussed by Alemán[135] *(*Scheme 7*)*. **Cat. B** forms an enamine 58 after reaction with the aldehyde 56, and 58 Subsequently forms the EDA 59 with alkyl bromide 55 (Scheme 32).

The Substrate scope study of this reaction provided yields between 70−90% for the bulk of the products 57 synthesised, with 72-91% *e.e.* and conversions of the reagents of up to 100%, providing an efficient and selective pathway for this reaction. However, for a few examples, the conversion rate was less than 5%. This is due to an unfortunate complication with flow chemistry. Due to the narrowness of the tubing, any precipitation formed will clog the reactor and halt the reaction. In this example, the researchers attempted to circumvent this precipitation by changing the concentration of the Substrate in solution, as increases to the concentration caused larger amounts of precipitation. Regardless, for these examples, the precipitation did not stop, and the reaction was abandoned. Other side effects of flow can also include over-irradiation, which can also occur in batch. This arises when a flow rate is set to lower than the optimal conditions, resulting in a longer residence time that can cause side-product formation. For example, there was a decrease in chemoselectivity of product 57 at residence times longer than 5 minutes, causing side product formation, however the *e.e.* of 57 was unaffected.

Kappe and Ötvös’ work provides both an interesting insight into certain drawbacks of flow, as well as some possible solutions, alongside a highly selective and efficient pathway for the enantioselective α-alkylation of aldehydes via an EDA pathway. The reaction required just a 5-minute residence time, corresponding to multigram per hour productivity, as compared to reaction times of up to 103 hours in batch, demonstrating a huge improvement in productivity and efficiency which easily outweighs the shortcomings of the precipitation issue for a few examples.

**Scheme 33:**
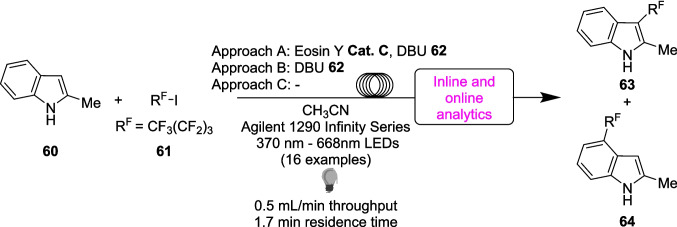
Outline of the perfluoroalkylation of 2-methylindole 60 performed in continuous flow, investigating a range of known photoredox catalyst and EDA-approaches in an array of 16 different LED wavelengths, utilising 3 different approaches [200]

Finally, Tallarek utilised a flow reactor for a less conventional approach, utilising the adaptability of LED arrays to conduct a screening of different wavelengths for both the photocatalyst and EDA-based methods for the perfluoroalkylation of 2-methylindole 60 (Scheme 33) [200]. These reactions pathways are known [80, 201–203] and have been studied primarily in batch, with vague descriptions of the wavelength of light used, such as “white light” and “blue LED”. These various reaction pathways have been combined in flow, accessing the different pathways by removing or adding eosin Y **Cat. C** and DBU 62. Three pathways have been devised, with eosin Y **Cat. C** and DBU 62 in a photocatalytic reaction (Approach A), removing eosin Y **Cat. C** and using DBU 62 in an EDA approach (Approach B), or removing both, leading to the formation of an EDA between 60 and 61 (Approach C). Approaches A-C were investigated with a range of sixteen different wavelengths within the same continuous flow manifold, as well as utilising a range of inline and online analytical techniques (See Figure 3) which allows for analytical monitoring during the reaction [119, 123] (Scheme 33). In this reaction, the researchers utilised an inline HPLC to measure progress of product formation, while an inline diode array detector (DAD) was used to verify whether the reactor was operating in steady state. However, using the definitions as laid out by Vilela (Figure 3) [129], the HPLC is better defined to be an atline reaction monitoring procedure, as the sample solution does not return to the reaction solution.

**Fig. 4:**
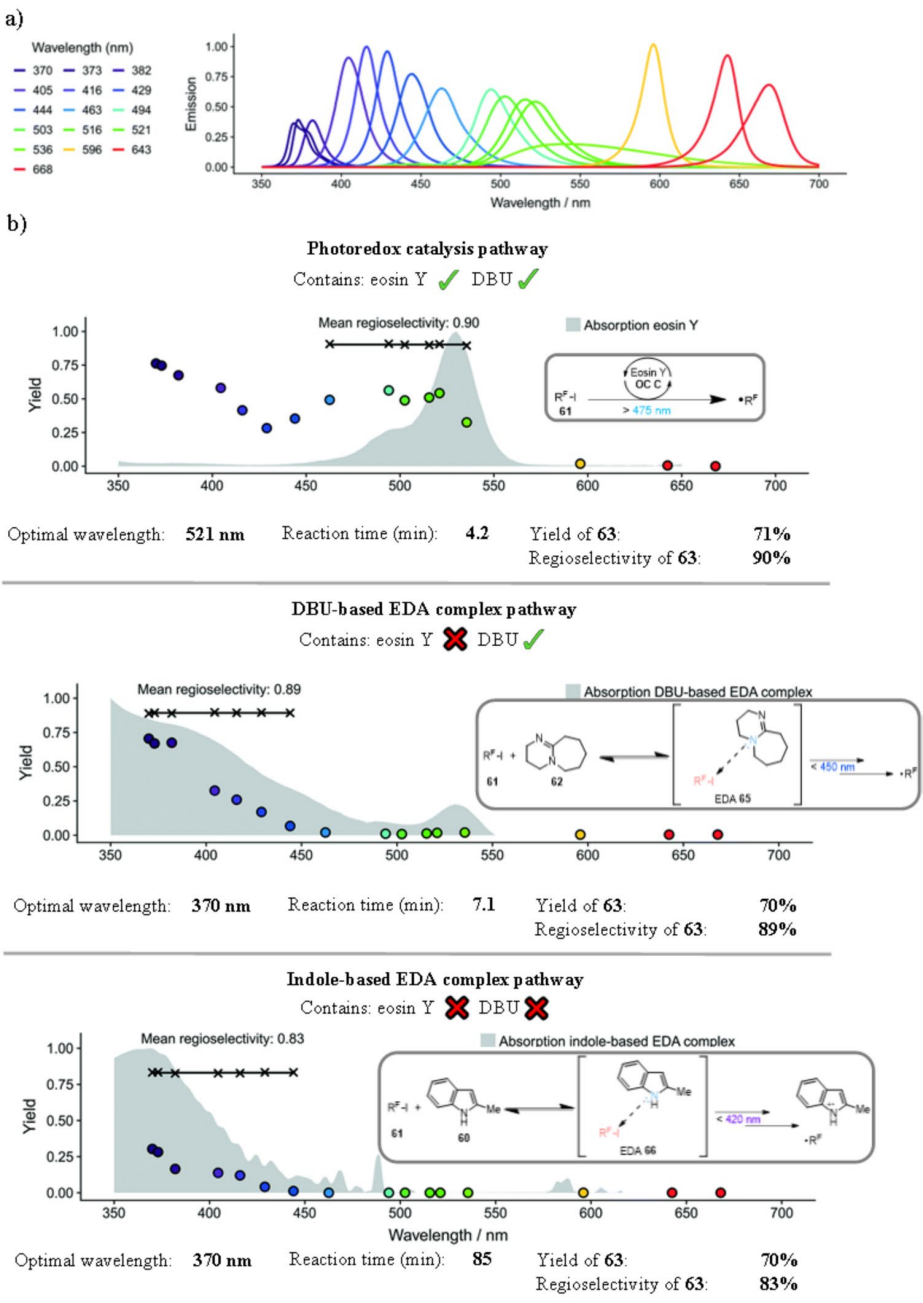
**A**) The emission spectra of the LED arrays. The transmission of the LEDs for wavelengths below 400nm decreased, reducing irradiation of the solution. For these examples, the reaction time was adjusted to compensate. **B**) Experimental results of the wavelength screening including yields of 49 and mechanisms of the radical production. Edited, Adapted and Reproduced from Haas, Roider, Hoffmann and Tallarek [200] with permission from the Royal Society of Chemistry, Creative Commons Attribution 3.0 Unported Licence [204]

The screening of irradiation wavelengths and intensities for photochemical reactions is not necessarily routine and systematic, but it can be used to successfully optimise photocatalyst or EDA reactivity. This was observed here, where longer wavelengths were optimal for photocatalysis while shorter wavelengths were more favourable for the EDA-pathways. This observation can be attributed to the different absorption spectra of eosin Y **Cat. C** and those of an EDA-complex (Figure 4). The screening of wavelengths also shows that EDA 65, formed in the reaction between nonaflouro-1-iodobutane 61 and DBU 62, was much more efficient in its formation compared to EDA 66. The reaction time for Approach B was almost 2 times faster than the pathway involving the indole 60 based EDA 66 (Approach C). Studies also found that the formation of EDA 65 was much more favourable when both indole 60 and DBU 61 where present.

The systematic testing of individual wavelengths of light is much more manageable in flow, as variables such as temperature, light intensity, reaction time and reaction throughput can be easily and manageably controlled in flow. It also highlights that more thorough irradiation screening is lacking in research of photochemistry, as opposed to other reaction parameters such as concentration, substrates or solvents. This research was also the only example in this review to demonstrate the use of reaction monitoring techniques, which, as discussed in Section "EDA photochemistry", allows for real-time monitoring and control capabilities, and enables immediate detection and monitoring of the progress of the reaction.

## Conclusion and Outlook

In this review, we have summarised the field of photochemical reactions containing EDA-complexes that have been developed or adapted for flow and discussed the reasons behind using flow chemistry for EDA-based photochemical reactions including for successful scale-up, to increase productivity and efficiency, as well as for systematic screening of various reaction parameters such as irradiation wavelengths and intensities. Continued research and development in this field will no doubt continue, especially as ever more EDA pathways are being discovered, while flow is also becoming a more established technique for conducting photochemical reactions.

EDA photochemistry is set to play a major role in photochemical reactions within flow in the future, as their ability to facilitate diverse chemical transformations under mild conditions, and without the need for an exogenous photocatalyst, aligns well with the principles of green chemistry. The relatively much lower costs of developing an EDA pathway as compared to the cost of a transition metal or organo- catalyst, should also be considered as a significant advantage for researchers working in this field, and transformations that use these catalysts should be studied for any potential alternative sustainable EDA pathway. As an example, proteins have been found to stabilise EDA complexes [71, 205], and so by combining photo-enzyme EDAs or protein stabilised EDAs with flow chemistry could open up and exciting new avenue for this field within biochemistry.

However, the use of flow, despite the aforementioned advantages, can also come with potential limitations, such as the risk of clogging if solids are present in the reaction mixture. Researchers have been able to overcome this issue by altering reagents to avoid any solid crashing out, but this solution is not always ideal. A potential area for future development would be to investigate techniques in flow which could overcome problems with clogging, for example, employing the use of a sonicator to break up solids within the reactor. A continuous stir tank reactor, or CSTR, is another potential method to overcome issues with clogging, however this negates one of the main advantages of photochemistry in flow; the uniform efficient irradiation of the solution in small tubing, rather than relying on a larger volume reaction vessel.

Additionally, future work could focus on the further utilisation of reaction monitoring techniques in flow, for example, a convenient and accurate method to monitor reactions inline, rather than offline, within the photochemical flow reactor itself. Due to the fact that UV-vis spectroscopy is the key spectroscopic technique in identifying an EDA, the utilisation of inline UV-vis spectroscopy within a flow set-up would be a useful technique to track the bathochromic shift, caused by the formation of the EDA, in real-time.

Future researchers can also capitalize on the simple and easily adjustable system design of flow. We have explored a wavelength scoping approach within Section "Exploring other merits and drawbacks of flow", which lead to the determination of the optimal wavelength for activating an EDA mechanism. This approach can be expanded, potentially using a computer-based analytic approach. A recent paper by de Oliveira has established an analytical approach to discover new EDA-mediated photochemical reactions by combining digital image processing and TLC analysis [206], and this could provide an interesting source of inspiration for a flow based approach for the development, screening and investigation of EDA complexes and mechanisms which have not previously been known.

## Data Availability

Not applicable. This is a review article and does not contain any original data.

## References

[1] Dincer I, Rosen MA (1999) Appl Energ:427-440

[2] Hammond GP (2000) Process Saf Environ 78:304–323

[3] Kalogirou SA (2004) Energ Convers Manage 45:3075–3092

[4] Jefferson M (2006) Renew. Energ. 31:571–582

[5] Omer AM (2008) Renew. Sust. Energ. Rev. 12:2265–2300

[6] Kaygusuz K (2009) Energ. Source. Part B 4:122–133

[7] Welch JB, Venkateswaran A (2009) Renew Sust Energ Rev 13:1121–1126

[8] Panwar NL, Kaushik SC, Kothari S (2011) Renew. Sust. Energ. Rev. 15:1513–1524

[9] Khan J, Arsalan MH (2016) Renew. Sust. Energ. Rev. 55:414–425

[10] Novas N, Garcia RM, Camacho JM, Alcayde A (2021) Sustain. 13:6295

[11] Jortner J (1976) J. Chem. Phys. 64:4860–4867

[12] Flynn JH (1990) J. of Therm. Anal. 36:1579–1593

[13] Zaman MH, Sosnick TR, Stephen Berry R (2003) Phys. Chem. Chem. Phys. 5:2589–2594

[14] Smith IW (2008) Chem. Soc. Rev. 37:812–826 18362985 10.1039/b704257b

[15] Aquilanti V, Mundim KC, Elango M, Kleijn S, Kasai T (2010) Chem. Phys. Lett. 498:209–213

[16] Ciamician G (1912) Science 36:385–394 17836492 10.1126/science.36.926.385

[17] Ciamician G, Silber P (1908) Ber Dtsch Chem Ges 41:1928–1935

[18] Prier CK, Rankic DA, MacMillan DW (2013) Chem Rev 113:5322–5363 23509883 10.1021/cr300503r PMC4028850

[19] Schultz DM, Yoon TP (2014) Science 343:1239176 24578578 10.1126/science.1239176 PMC4547527

[20] Shaw MH, Twilton J, MacMillan DW (2016) J Org Chem 81:6898–6926 27477076 10.1021/acs.joc.6b01449 PMC4994065

[21] Staveness D, Bosque I, Stephenson CR (2016) Accounts Chem. Res. 49:2295–2306 10.1021/acs.accounts.6b00270 PMC5127252 27529484

[22] Yoon TP, Ischay MA, Du J (2010) Nat. Chem. 2:527–532 20571569 10.1038/nchem.687

[23] Oelgemöller M (2014) J Chin Chem Soc 61:743–748

[24] Trommsdorff H (1834) Ann Phar 11:190–207

[25] Natarajan A, Tsai CK, Khan SI, McCarren P, Houk KN, Garcia-Garibay MA (2007) J Am Chem Soc 129:9846–9847 17645337 10.1021/ja073189o

[26] Romero NA, Nicewicz DA (2016) Chem Rev 116:10075–10166 27285582 10.1021/acs.chemrev.6b00057

[27] Lang X, Chen X, Zhao J (2014) Chem Soc Rev 43:473–486 24162830 10.1039/c3cs60188a

[28] Nicewicz DA, Nguyen TM (2013) ACS Catal. 4:355–360

[29] Murphy JJ, Melchiorre P (2015) Nature 524:297–298 26266981 10.1038/nature15200

[30] Yoon TP (2013) ACS Catal 3:895–902 23691491 10.1021/cs400088e PMC3656437

[31] Behm K, McIntosh RD (2020) ChemPlusChem 85:2611–2618 33263950 10.1002/cplu.202000610

[32] Institute AGC (2024) Endangered elements. https://www.acs.org/greenchemistry/research-innovation/endangered-elements.html. Cited 19-08-2024

[33] Cole-Hamilton DJ (2019) Chem. Int. 41:23–28

[34] Bobo MV, Kuchta JJ III, Vannucci AK (2021) Org. Biomol. Chem. 19:4816–4834 34008685 10.1039/d1ob00396h

[35] Hari DP, König B (2014) Chem. Commun. 50:6688–6699 10.1039/c4cc00751d 24699920

[36] Neumann M, Fuldner S, Konig B, Zeitler K (2011) Angew. Chem. Int. Edit. 50:951–954 10.1002/anie.201002992 20878819

[37] Heitz DR, Rizwan K, Molander GA (2016) J. Org. Chem. 81:7308–7313 27336284 10.1021/acs.joc.6b01207 PMC4994715

[38] MacMillan DW (2008) Nature 455:304–308 18800128 10.1038/nature07367

[39] Twilton J, Le C, Zhang P, Shaw MH, Evans RW, MacMillan DWC (2017) Nat. Rev. Chem. 1:0052

[40] Wallentin CJ, Nguyen JD, Stephenson CR (2012) Chimia 66:394–398 22871282 10.2533/chimia.2012.394

[41] Narayanam JM, Stephenson CR (2011) Chem. Soc. Rev. 40:102–113 20532341 10.1039/b913880n

[42] Skubi KL, Yoon TP (2014) Nature 515:45–46 25373672 10.1038/515045a

[43] Nicewicz DA, MacMillan DW (2008) Science 322:77–80 18772399 10.1126/science.1161976 PMC2723798

[44] Pistritto VA, Liu S, Nicewicz DA (2022) J. Am. Chem. Soc. 144:15118–15131 35944280 10.1021/jacs.2c04577 PMC10037305

[45] Perkowski AJ, Cruz CL, Nicewicz DA (2015) J. Am. Chem. Soc. 137:15684–15687 26645387 10.1021/jacs.5b11800

[46] Chen W, Wang H, Tay NES, Pistritto VA, Li KP, Zhang T, Wu Z, Nicewicz DA, Li Z (2022) Nat Chem 14:216–223 34903859 10.1038/s41557-021-00835-7 PMC9617144

[47] Buzzetti L, Crisenza GEM, Melchiorre P (2019) Angew. Chem. Int. Edit. 58:3730–3747 10.1002/anie.201809984 30339746

[48] Le Saux E, Ma D, Bonilla P, Holden CM, Lustosa D, Melchiorre P (2021) Angew. Chem. Int. Edit. 60:5357–5362 10.1002/anie.202014876 PMC7986922 33283919

[49] Mazzarella D, Crisenza GEM, Melchiorre P (2018) J Am Chem Soc 140:8439–8443 29932655 10.1021/jacs.8b05240

[50] Foster R (1980) J Phys Chem 84:2135–2141

[51] Mulliken RS (1950) J Am Chem Soc 72:600–608

[52] Mulliken RS (1952) J Am Chem Soc 74:811–824

[53] Mulliken RS (1952) J Phys Chem 56:801–822

[54] Wortman AK, Stephenson CRJ (2023) Chem 9:2390–2415 37873033 10.1016/j.chempr.2023.06.013 PMC10588808

[55] Crisenza GEM, Mazzarella D, Melchiorre P (2020) J Am Chem Soc 142:5461–5476 32134647 10.1021/jacs.0c01416 PMC7099579

[56] Tasnim T, Ayodele MJ, Pitre SP (2022) J Org Chem 87:10555–10563 35904501 10.1021/acs.joc.2c01013

[57] Volkov AA, Bugaenko DI, Karchava AV (2024) ChemCatChem 16:e202301526

[58] Yerien DE, Barata-Vallejo S, Postigo A (2024). ChemPhotoChem. 10.1002/cptc.202400112

[59] Rehm TH (2020) Chem. Eur. J. 26:16952–16974 32427387 10.1002/chem.202000381 PMC7821313

[60] Xie J, Zhao D (2020) Chinese Chem. Lett. 31:2395–2400

[61] Thomson CG, Lee AL, Vilela F (2020) Beilstein J Org Chem 16:1495–1549 32647551 10.3762/bjoc.16.125 PMC7323633

[62] Politano F, Oksdath-Mansilla G (2018) Org Process Res Dev 22:1045–1062

[63] Plutschack MB, Pieber B, Gilmore K, Seeberger PH (2017) Chem Rev 117:11796–11893 28570059 10.1021/acs.chemrev.7b00183

[64] Noël T (2017) J Flow Chem 7:87–93

[65] Cambie D, Bottecchia C, Straathof NJ, Hessel V, Noel T (2016) Chem Rev 116:10276–10341 26935706 10.1021/acs.chemrev.5b00707

[66] Sambiagio C, Noël T (2020) Trends Chem. 2:92–106

[67] Prout CK, Wright JD (1968) Angew. Chem. Int. Edit. 7:659–667

[68] Wortman AK, Stephenson CR (2023) Elsevier. Copyright Clearance Centre

[69] Masuhara H, Mataga N (1972) B. Chem. Soc. Jpn. 45:43–47

[70] Arimitsu S, Tsumbomura H (1972) B. Chem. Soc. Jpn. 45:1357–1361

[71] Rosokha SV, Kochi JK (2008) Accounts Chem. Res. 41:641–653 10.1021/ar700256a 18380446

[72] Piedra HF, Tagarro I, Plaza M (2025) Org Chem Front 12:3920–3941

[73] Konovalova TA, Volodin AM (1993) React Kinet Catal Lett 51:227–232

[74] Kochi JK (1991) Pure Appl Chem 63:255–264

[75] Hamity M, Lema RH (1988) Can J Chem 66:1552–1557

[76] de Pedro Beato E, Spinnato D, Zhou W, Melchiorre P (2021) J Am Chem Soc 143:12304–12314 34320312 10.1021/jacs.1c05607 PMC8361436

[77] Zhou W, Wu S, Melchiorre P (2022) J Am Chem Soc 144:8914–8919 35549337 10.1021/jacs.2c03546

[78] Yoshihara K, Futamura K, Nagakura S (1972) Chem. Lett. 1:1243–1247

[79] Nappi M, Bergonzini G, Melchiorre P (2014) Angew. Chem. Int. Edit. 53:4921–4925 10.1002/anie.201402008 24668827

[80] Kandukuri SR, Bahamonde A, Chatterjee I, Jurberg ID, Escudero-Adan EC, Melchiorre P (2015) Angew. Chem. Int. Edit. 54:1485–1489 10.1002/anie.201409529 25475488

[81] Saxena B, Patel RI, Sharma A (2023) Adv Synth Catal 365:1538–1564

[82] Job P (1928) Ann. Chim. 9:113–134

[83] Basha MT, Alghanmi RM, Soliman SM, Alharby WJ (2020) J Mol Liq 309

[84] Al-Ahmary KM, Habeeb MM, Al-Solmy EA (2011) J Mol Liq 162:129–134

[85] Neelgund GM, Kulkarni AS, Budni ML (2004) Monatsh. Chem. 135:343–355

[86] Renny JS, Tomasevich LL, Tallmadge EH, Collum DB (2013) Angew. Chem. Int. Edit. 52:11998–12013 10.1002/anie.201304157 PMC4028694 24166797

[87] Guadalupe Hernández J, Huerta-Aguilar CA, Thangarasu P, Höpfl H (2017) New J Chem 41:10815–10827

[88] Yabe T, Kochi JK (1992) J Am Chem Soc 114:4491–4500

[89] Bockman TM, Kochi JK (1989) J Am Chem Soc 111:4669–4683

[90] Jarzeba W, Thakur K, Hörmann A, Barbara PF (1995) J Phys Chem 99:2016–2023

[91] Huyskens PL, Vael-Pauwels C, Seghers K (1992) B. Soc. Chim. Belg. 101:449–462

[92] Bockman TM, Karpinski ZJ, Sankararaman S, Kochi JK (1992) J Am Chem Soc 114:1970–1985

[93] Kochi JK (1994) Adv. Phys. Org. Chem. 29:185–272

[94] Ma L, Li J, Zhang X, Yang Y, Lin X, Chen X (2024) J Phys Chem Lett 15:3412–3418 38502941 10.1021/acs.jpclett.4c00455

[95] Yang Z, Liu Y, Cao K, Zhang X, Jiang H, Li J (2021) Beilstein J. Org. Chem. 17:771–799 33889219 10.3762/bjoc.17.67 PMC8042489

[96] Zheng L, Cai L, Tao K, Xie Z, Lai YL, Guo W (2021) Asian. J. Org. Chem. 10:711–748

[97] Yuan Y-Q, Majumder S, Yang M-H, Guo S-R (2020) Tetrahedron Lett. 61:151506

[98] Hyeon Ka C, Kim S, Jin Cho E (2023) Chem. Rec. 23:e202300036 36942971 10.1002/tcr.202300036

[99] Bhanja R, Bera SK, Mal P (2023) Adv. Synth. Catal. 366:168–182

[100] Whitesides GM (2006) Nature 442:368–373 16871203 10.1038/nature05058

[101] Milorad PD, Faical L, Patrick LM (1999) Chem. Eng. Sci. 54:1975–1995

[102] Wegner J, Ceylan S, Kirschning A (2011) Chem Commun 47:4583–4592 10.1039/c0cc05060a 21409184

[103] Goršek A, Glavič P (1997) Chem. Eng. Res. Des. 75:709–717

[104] Löwe H, Ehrfeld W (1999) Electrochim Acta 44:3679–3689

[105] Johnson MD, Braden T, Calvin JR, Campbell Brewer A, Cole KP, Frank S, Kerr M, Kjell D, Kopach ME, Martinelli JR, May SA, Rincon J, White TD, Yates MH (2023) Chimia 77:319-326 10.2533/chimia.2023.319 38047828

[106] Porta R, Benaglia M, Puglisi A (2015) Org. Process Res. Dev. 20:2–25

[107] Su Y, Straathof NJ, Hessel V, Noel T (2014) Chem. Eur. J. 20:10562–10589 25056280 10.1002/chem.201400283

[108] Knowles JP, Elliott LD, Booker-Milburn KI (2012) Beilstein J. Org. Chem. 8:2025–2052 23209538 10.3762/bjoc.8.229 PMC3511038

[109] Garlets ZJ, Nguyen JD, Stephenson CR (2014) Isr. J. Chem. 54:351–360 25484447 10.1002/ijch.201300136 PMC4255365

[110] Ahmed-Omer B, Barrow DA, Wirth T (2009) Tetrahedron Lett 50:3352–3355

[111] Naber JR, Buchwald SL (2010) Angew. Chem. Int. Edit. 49:9469–9474 10.1002/anie.201004425 21038337

[112] Ceylan S, Coutable L, Wegner J, Kirschning A (2011) Chem. Eur. J. 17:1884–1893 21274939 10.1002/chem.201002291

[113] Ceylan S, Friese C, Lammel C, Mazac K, Kirschning A (2008) Angew. Chem. Int. Edit. 47:8950–8953 10.1002/anie.200801474 18924199

[114] Nijhuis TA, van Koten G, Kapteijn F, Moulijn JA (2003) Catal. Today 79–80:315–321

[115] Fischer M (1978) Angew. Chem. Int. Edit. 17:16–26

[116] Beer A (1852) Ann Phys-Leipzig 162:1-160

[117] Hook BDA, Dohle W, Hirst PR, Pickworth M, Berry MB, Booker-Milburn KI (2005) J. Org. Chem. 70:7459–7798 16149784 10.1021/jo050705p

[118] Talla A, Driessen B, Straathof NJW, Milroy LG, Brunsveld L, Hessel V, Noël T (2015) Adv Synth Catal 357:2180–2186

[119] Foley DA, Wang J, Maranzano B, Zell MT, Marquez BL, Xiang Y, Reid GL (2013) Anal Chem 85:8928–8932 24003862 10.1021/ac402382d

[120] Schwolow S, Braun F, Rädle M, Kockmann N, Röder T (2015) Org Process Res Dev 19:1286–1292

[121] Gomez MV, de la Hoz A (2017) Beilstein J Org Chem 13:285–300 28326137 10.3762/bjoc.13.31 PMC5331343

[122] Galaverna R, Ribessi RL, Rohwedder JJR, Pastre JC (2018) Org Process Res Dev 22:780–788

[123] Giraudeau P, Felpin F-X (2018) React. Chem. Eng. 3:399–413

[124] Fonseca GE, Dubé MA, Penlidis A (2009) Macromol. React. Eng. 3:327–373

[125] Frauendorfer E, Wolf A, Hergeth WD (2010) Chem. Eng. Technol. 33:1767–1778

[126] Knox ST, Warren NJ (2020) React. Chem. Eng. 5:405–423

[127] Colegrove B, Deshpande K, Harner R, Mikolajczyk L, Stephenson SK, Tate JD, Weston J (2017) Macromol. React. Eng. 11:1600056

[128] Alb AM, Reed WF (2010) Macromol. React. Eng. 4:470–485

[129] Patterson SBH, Wong R, Barker G, Vilela F (2023) J Flow Chem 13:103–119

[130] Commons C (2024) Attribution 4.0 international. https://creativecommons.org/licenses/by/4.0/. Cited 14-08-2024

[131] Patterson SBH, Arrighi V, Vilela F (2024) ACS Macro Lett. 13:508–514 38625870 10.1021/acsmacrolett.4c00117 PMC11112751

[132] McClain EJ, Monos TM, Mori M, Beatty JW, Stephenson CRJ (2020) ACS Catal. 10:12636–12641

[133] Lee KY, Kochi JK (1992) J Chem Soc Perkin Trans 2. 10.1039/P29920001011:1011-1017

[134] Uniqsis Ltd (2024) Uniqsis ltd - photosyn™. https://www.uniqsis.com/paProductsDetail.aspx?ID=PhotoSyn. Cited 16-08-2024

[135] Rodriguez RI, Sicignano M, Aleman J (2022) Angew. Chem. Int. Edit. 61:e202112632 10.1002/anie.202112632 34982505

[136] Chipperfield MP, Hossaini R, Montzka SA, Reimann S, Sherry D, Tegtmeier S (2020) Nat. Rev. Earth Environ. 1:251–263

[137] Aziz J, Messaoudi S, Alami M, Hamze A (2014) Org. Biomol. Chem. 12:9743–9759 25354469 10.1039/c4ob01727g

[138] Reddy RJ, Kumari AH (2021) RSC Adv. 11:9130–9221 35423435 10.1039/d0ra09759d PMC8695481

[139] Vapourtec Ltd (2024) Photochemical reactions uv-150 reactor. https://www.vapourtec.com/products/flow-reactors/photochemical-reactor-uv-150-features/. Cited 16-08-2024

[140] Gavory G, O’Dowd CR, Helm MD, Flasz J, Arkoudis E, Dossang A, Hughes C, Cassidy E, McClelland K, Odrzywol E, Page N, Barker O, Miel H, Harrison T (2018) Nat. Chem. Biol. 14:118–125 29200206 10.1038/nchembio.2528

[141] O’Dowd CR, Helm MD, Rountree JSS, Flasz JT, Arkoudis E, Miel H, Hewitt PR, Jordan L, Barker O, Hughes C, Rozycka E, Cassidy E, McClelland K, Odrzywol E, Page N, Feutren-Burton S, Dvorkin S, Gavory G, Harrison T (2018) ACS Med. Chem. Lett. 9:238–243 29541367 10.1021/acsmedchemlett.7b00512 PMC5846043

[142] Batista GMF, de Castro PP, Dos Santos HF, de Oliveira KT, Amarante GW (2020) Org. Lett. 22:8598–8602 33086786 10.1021/acs.orglett.0c03187

[143] Zhu L, Wang LS, Li B, Fu B, Zhang CP, Li W (2016) Chem. Commun. 52:6371–6374 10.1039/c6cc01944g 26996326

[144] Wang H, Zhang J, Shi J, Li F, Zhang S, Xu K (2019) Org. Lett. 21:5116–5120 31247795 10.1021/acs.orglett.9b01714

[145] Rosso C, Williams JD, Filippini G, Prato M, Kappe CO (2019) Org. Lett. 21:5341–5345 31247766 10.1021/acs.orglett.9b01992

[146] Barata-Vallejo S, Cooke MV, Postigo A (2018) ACS Catal. 8:7287–7307

[147] Abderrazak Y, Reiser O (2024) ACS Catal. 14:4847–4855

[148] Nguyen JD, Tucker JW, Konieczynska MD, Stephenson CRJ (2011) J. Am. Chem. Soc. 133:4160–4163 21381734 10.1021/ja108560e PMC3086499

[149] Wallentin C-J, Nguyen JD, Finkbeiner P, Stephenson CRJ (2012) J. Am. Chem. Soc. 134:8875–8884 22486313 10.1021/ja300798k

[150] Arceo E, Montroni E, Melchiorre P (2014) Angew. Chem. Int. Edit. 53:12064–12068 10.1002/anie.201406450 25243823

[151] Yajima T, Ikegami M (2017) Eur. J. Org. Chem. 2017:2126–2129

[152] Beniazza R, Abadie B, Remisse L, Jardel D, Lastecoueres D, Vincent JM (2017) Chem. Commun. 53:12708–12711 10.1039/c7cc05854c 29138763

[153] Wang Y, Wang J, Li G-X, He G, Chen G (2017) Org. Lett. 19:1442–1445 28263075 10.1021/acs.orglett.7b00375

[154] Rosso C, Filippini G, Cozzi PG, Gualandi A, Prato M (2019) ChemPhotoChem 3:193–197

[155] Krech A, Yakimchyk V, Jarg T, Kananovich D, Ošeka M (2023) Adv. Synth. Catal. 366:91–100

[156] Capaldo L, Buzzetti L, Merli D, Fagnoni M, Ravelli D (2016) J Org Chem 81:7102–7109 27322957 10.1021/acs.joc.6b00984

[157] Montanaro S, Ravelli D, Merli D, Fagnoni M, Albini A (2012) Org Lett 14:4218–4221 22852829 10.1021/ol301900p

[158] Xia Q, Li Y, Wang X, Dai P, Deng H, Zhang WH (2020) Org Lett 22:7290–7294 32902295 10.1021/acs.orglett.0c02631

[159] Kouznetsov VV, Galvis CEP (2018) Tetrahedron 74:773–810

[160] Rueping M, Zhu S, Koenigs RM (2011) Chem. Commun. 47:12709–12711 10.1039/c1cc15643h 22041859

[161] Ide T, Barham JP, Fujita M, Kawato Y, Egami H, Hamashima Y (2018) Chem. Sci. 9:8453–8460 30542595 10.1039/c8sc02965b PMC6244453

[162] Tian H, Xu W, Liu Y, Wang Q (2019) Chem. Commun. 55:14813–14816 10.1039/c9cc08056b 31763630

[163] Li Z, Ma P, Tan Y, Liu Y, Gao M, Zhang Y, Yang B, Huang X, Gao Y, Zhang J (2020) Green Chem. 22:646–650

[164] Xia Q, Li Y, Cheng L, Liang X, Cao C, Dai P, Deng H, Zhang W, Wang Q (2020) Org. Lett. 22:9638–9643 33285068 10.1021/acs.orglett.0c03703

[165] Seayad AM, Ramalingam B, Yoshinaga K, Nagata T, Chai CLL (2009) Org. Lett. 12:264–267 10.1021/ol902540h 20025268

[166] Strecker A (1850) Liebigs Ann. Chem. 75:27–45

[167] Han W, Ofial AR (2009) Chem Commun. 10.1039/b910548d:5024-5026 10.1039/b910548d 19668837

[168] Orejarena Pacheco JC, Lipp A, Nauth AM, Acke F, Dietz JP, Opatz T (2016) Chem. Eur. J. 22:5409–5415 26929114 10.1002/chem.201504845

[169] Kamijo S, Hoshikawa T, Inoue M (2011) Org Lett 13:5928–5931 21992181 10.1021/ol202659e

[170] Pan Y, Wang S, Kee CW, Dubuisson E, Yang Y, Loh KP, Tan C-H (2011) Green Chem. 13:3341–3344

[171] Lapworth A (1904) J. Chem. Soc. Faraday T. 85:1206–1214

[172] Liu MT, Liu DG, Qin ZW, Wang GZ (2022) Asian. J. Org. Chem. 11:e202200335

[173] Dondi D, Protti S, Albini A, Carpio SM, Fagnoni M (2009) Green Chem. 11:1653–1659

[174] McTiernan CD, Pitre SP, Scaiano JC (2014) ACS Catal. 4:4034–4039

[175] Li H, Tang X, Pang JH, Wu X, Yeow EKL, Wu J, Chiba S (2021) J Am Chem Soc 143:481–487 33356206 10.1021/jacs.0c11968

[176] Pauling L (1932) J Am Chem Soc 54:3570–3582

[177] Fu M-C, Shang R, Zhao B, Wang B, Fu Y (2019) Science 363:1429–1434 30923218 10.1126/science.aav3200

[178] Cuadros S, Rosso C, Barison G, Costa P, Kurbasic M, Bonchio M, Prato M, Filippini G, Dell’Amico L (2022) Org Lett 24:2961–2966 35437017 10.1021/acs.orglett.2c00604 PMC9062880

[179] d’Ischia M, Napolitano A, Ball V, Chen C-T, Buehler MJ (2014) Accounts Chem. Res. 47:3541–3550 10.1021/ar500273y 25340503

[180] Davin LB, Jourdes M, Patten AM, Kim KW, Vassao DG, Lewis NG (2008) Nat Prod Rep 25:1015–1090 19030603 10.1039/b510386j

[181] McGrath NA, Brichacek M, Njardarson JT (2010) J Chem Educ 87:1348–1349

[182] Quideau S, Deffieux D, Douat-Casassus C, Pouysegu L (2011) Angew Chem Int Edit 50:586–621 10.1002/anie.201000044 21226137

[183] Dai J-L, Shao N-Q, Zhang J, Jia R-P, Wang D-H (2017) J Am Chem Soc 139:12390–12393 28805383 10.1021/jacs.7b06785

[184] Huang Z, Lumb J-P (2019) ACS Catal 9:521–555

[185] Youn SW, Cho CG (2021) Org Biomol Chem 19:5028–5047 34027964 10.1039/d1ob00506e

[186] Filippini G, Nappi M, Melchiorre P (2015) Tetrahedron 71:4535–4542

[187] Ishikawa T, Kumamoto T (2006) Synth. 2006:737–752

[188] Kuivila HG, Nahabedian KV (1961) J Am Chem Soc 83:2159–2163

[189] Ono K, Erhard A (2011) Nondestructive testing, 3. Ultrasonics. In: Ullmann F, Ley C, Elvers B (eds) Ullmann's encyclopedia of industrial chemistry, 7th ed, Wiley, New York

[190] Sun B, Lv C, Zhuang X, Xu Y, Song H, Wang J, Zhang Z, Wang J, Jin C (2024) Green Chem 26:11531–11539

[191] Vitaku E, Smith DT, Njardarson JT (2014) J Med Chem 57:10257–10274 25255204 10.1021/jm501100b

[192] Kerru N, Gummidi L, Maddila S, Gangu KK, Jonnalagadda SB (2020) Molecules 25:1909 32326131 10.3390/molecules25081909 PMC7221918

[193] Shevlin M, Guan X, Driver TG (2017) ACS Catal 7:5518–5522

[194] Pocock E, Diefenbach M, Hood TM, Nunn M, Richards E, Krewald V, Webster RL (2024) J Am Chem Soc 146:19839–19851 38995168 10.1021/jacs.4c02797 PMC11273354

[195] Paolillo JM, Duke AD, Gogarnoiu ES, Wise DE, Parasram M (2023) J. Am. Chem. Soc. 145:2794–2799 36696364 10.1021/jacs.2c13502 PMC10032565

[196] Lasso JD, Castillo-Pazos DJ, Sim M, Barroso-Flores J, Li CJ (2023) Chem. Sci. 14:525–532 36741536 10.1039/d2sc06087f PMC9847664

[197] Wang B, Ren H, Cao HJ, Lu C, Yan H (2022) Chem. Sci. 13:11074–11082 36320483 10.1039/d2sc03590a PMC9516892

[198] Molnar M, Kappe CO, Otvos SB (2023) Adv. Synth. Catal. 365:1660–1670 38515505 10.1002/adsc.202300289 PMC10952295

[199] Arceo E, Jurberg ID, Alvarez-Fernandez A, Melchiorre P (2013) Nat. Chem. 5:750–756 23965676 10.1038/nchem.1727

[200] Haas CP, Roider T, Hoffmann RW, Tallarek U (2019) React. Chem. Eng. 4:1912–1916

[201] Gao GL, Yang C, Xia W (2017) Chem. Commun. 53:1041–1044 10.1039/c6cc08975e 28044149

[202] Wozniak L, Murphy JJ, Melchiorre P (2015) J. Am. Chem. Soc. 137:5678–5681 25901659 10.1021/jacs.5b03243 PMC4428001

[203] Straathof NJW, van Osch DJGP, Schouten A, Wang X, Schouten JC, Hessel V, Noël T (2014) J Flow Chem 4:12–17

[204] Commons C (2024) Attribution 3.0 unported. https://creativecommons.org/licenses/by/3.0/. Cited 14-08-2024

[205] Beil SB, Bonnet S, Casadevall C, Detz RJ, Eisenreich F, Glover SD, Kerzig C, Naesborg L, Pullen S, Storch G, Wei N, Zeymer C (2024) J. Am. Chem. Soc. 4:2746–2766 10.1021/jacsau.4c00527 PMC11350580 39211583

[206] Silva RC, Batista GMF, Brocksom TJ, de Oliveira KT (2024) Adv. Synth Catal 367

